# Genetic Associations with Type 2 Diabetes Differ According to Metabolic Contexts Defined by Insulin Resistance and Visceral Adiposity

**DOI:** 10.3390/ijms27219844

**Published:** 2026-11-03

**Authors:** Bo-Kyung Shine, Kyung-Won Hong, Ja-Eun Choi, Bom Taeck Kim, Yu-Jin Kwon, In Hwa Jeong

**Affiliations:** 1 Department of Family Medicine, College of Medicine, Dong-A University, Busan 49201, Republic of Korea; sbk123@dau.ac.kr; 2 Theragen Health Co., Ltd., Yongin 16976, Republic of Korea; kyungwon.hong@theragenhealth.com (K.-W.H.); jaeun.choi@theragenhealth.com (J.-E.C.); 3 Department of Family Practice and Community Health, Ajou University School of Medicine, Suwon 16499, Republic of Korea; lovesong@ajou.ac.kr; 4 Department of Family Medicine, Severance Hospital, Yonsei University College of Medicine, Seoul 03722, Republic of Korea; digda3@yuhs.ac; 5 Department of Laboratory Medicine, Dong-A University Hospital, Dong-A University College of Medicine, 26 Daesingongwon-ro, Seo-gu, Busan 49201, Republic of Korea

**Keywords:** diabetes, genome-wide association study, metabolic score for insulin resistance, metabolic score for visceral fat, visceral adiposity, genetic susceptibility, Korean Genome and Epidemiology Study

## Abstract

Stratifying populations based on indicators of insulin resistance and visceral adiposity may help identify genetic associations with type 2 diabetes mellitus (T2DM) that vary across metabolic environments. This study aimed to investigate metabolic context-dependent genetic associations with T2DM using the metabolic score for insulin resistance (METS-IR) and visceral fat (METS-VF). A total of 70,583 and 70,513 participants were eligible for METS-IR and METS-VF stratification, respectively. After excluding participants with missing diabetes status, the final case–control samples comprised 40,970 and 40,944 participants, respectively. Genome-wide association studies of prevalent T2DM were conducted within metabolic tertiles, with additional evaluation of lead variants in the intermediate tertile (T2). Logistic regression under an additive genetic model was performed, followed by genotype × metabolic stratum interaction analyses and Cochran’s Q heterogeneity tests. Twelve notable loci were identified, showing both shared and varying association patterns across metabolic strata. *CDKAL1* showed robust associations across metabolic strata, with progressively attenuated effect sizes from T1 to T3 and significant interaction results under both METS-VF and METS-IR stratifications. *PAX4*, *CPEB3*, and *HHEX* also demonstrated stronger associations in metabolically favorable groups, supported by nominally significant interaction and heterogeneity tests. *CDKN2A/B* showed associations across metabolic strata, with evidence of differences in genetic effects. *KCNQ1* exhibited broadly consistent associations, although evidence of interaction varied by metabolic index. *CNNM2*, *HNF1B*, *HNF4A*, *SLC30A8*, and *DNAJC5B–TRIM55* displayed subgroup-dependent patterns of statistical significance, which did not uniformly correspond to significant interaction effects. *ALDH2* showed distinct association patterns across metabolic strata and between men and women. These findings suggest that genetic associations with T2DM may vary according to metabolic context, although subgroup-specific genome-wide significance does not necessarily establish differential genetic effects. Formal interaction and heterogeneity analyses provide additional evidence for metabolic effect modification at selected loci, suggesting that metabolic stratification may help characterize variation in genetic associations with T2DM in the Korean population.

## 1. Introduction

Type 2 diabetes mellitus (T2DM) arises from a complex interplay between inherited genetic susceptibility and environmental stressors [1]. Over the past two decades, genome-wide association studies (GWAS) have identified numerous T2DM susceptibility loci. These loci consistently demonstrate an enrichment in pathways related to pancreatic β-cell dysfunction and impaired insulin secretion rather than peripheral insulin resistance [2]. However, T2DM is phenotypically heterogeneous, and individuals with comparable genetic risk follow vastly different metabolic trajectories [3]. Correspondingly, conventional GWAS approaches based on broadly defined case–control status often fail to capture context-dependent genetic effects. Therefore, stratifying populations according to specific metabolic environments may improve the detection of biologically meaningful genetic associations [4].

Insulin resistance [5] and visceral adiposity [6] are two closely related yet biologically distinct drivers of T2DM. The metabolic score for insulin resistance (METS-IR) serves as a reliable surrogate index of systemic insulin resistance [7]. Conversely, the metabolic score for visceral fat (METS-VF) estimates visceral adiposity, which is a major contributor to lipotoxicity and chronic low-grade inflammation [8]. Direct assessment of insulin resistance using the hyperinsulinemic–euglycemic clamp and quantification of visceral adipose tissue using computed tomography or magnetic resonance imaging are resource-intensive and often impractical in large population-based cohorts. In contrast, METS-IR and METS-VF can be calculated from routinely collected anthropometric and biochemical measurements without requiring specialized procedures. Previous validation studies have demonstrated that METS-IR correlates with established measures of insulin sensitivity, while METS-VF provides an estimate of visceral adiposity supported by imaging-based assessments [7,8]. These characteristics make both indices practical surrogate measures for characterizing metabolic heterogeneity in large-scale genetic epidemiological studies. Although both indices predict cardiometabolic risk, they represent distinct biological contexts. In particular, systemic insulin resistance is influenced by various dynamic factors [5], including lifestyle behaviors, medication use, and disease progression. In contrast, visceral adiposity reflects a persistent metabolic state characterized by lipotoxicity, chronic inflammation, and ectopic fat accumulation [9]. Despite the extensive volume of T2DM GWASs, few studies have evaluated variation in genetic signals across metabolic environments as defined by these surrogate indices.

In this methodological context, East Asian populations provide a critical and highly informative setting for exploring context-specific genetic architecture. Specifically, East Asian populations frequently develop T2DM at lower body mass index (BMI) levels than those of Western cohorts. This is primarily due to a lower baseline insulin secretory capacity in East Asian populations relative to their degree of insulin resistance [10]. Previous studies have highlighted the potential contribution of population-specific genetic variants to T2DM susceptibility. For example, a study conducted in a North Indian population identified a significant association between the NLRP3 rs10754558 polymorphism and T2DM, whereas the PRKCZ rs2503706 polymorphism was not significantly associated with the disease. These findings suggest that genetic susceptibility to T2DM may involve inflammation-related pathways in addition to established mechanisms of pancreatic β-cell dysfunction and insulin resistance [11]. In this regard, several T2DM-associated loci, such as variants near *PAX4* [12], *KCNQ1* [13], and *ALDH2* [14,15], exhibit population-specific allele frequencies and distinct biological relevance in East Asian populations. These unique pathophysiological and genetic characteristics strongly suggest that stratified GWASs within the Korean population can provide critical insights into context-dependent genetic mechanisms that remain obscured in broader unstratified analyses.

Accordingly, this study analyzed approximately 70,000 participants from the Korean Genome and Epidemiology Study (KoGES). Specifically, GWASs were conducted to identify genetic associations with prevalent T2DM across subgroups defined by tertiles of METS-IR and METS-VF in the total population and sex-stratified subgroups. Overall, the study aimed to identify robust and potentially context-dependent susceptibility loci for T2DM and explore sex-stratified association patterns by comparing genetic associations across contrasting metabolic strata. Through this approach, the study aimed to provide a nuanced understanding of the genetic architecture of T2DM across distinct metabolic environments. Furthermore, the study aimed to explore the influence of the metabolic context on the detectability of genetic susceptibility loci.

## 2. Results

### 2.1. Baseline Characteristics of the Study Population

The initial populations eligible for metabolic stratification comprised 70,513 participants for METS-VF and 70,583 participants for METS-IR. After excluding participants with missing diabetes status, the final case–control samples comprised 40,944 and 40,970 participants, respectively. To establish contrasting metabolic environments, the participants were independently stratified into tertiles based on each metabolic score, with the lowest (T1) and highest (T3) tertiles used for subsequent downstream analyses. The streamlined clinical, biochemical, and lifestyle characteristics of the study participants across these strata are summarized in Table 1. The distributions of T2DM cases and controls across METS-IR and METS-VF tertiles, stratified by sex, are presented in Supplementary Table S1. 

**Table 1:** Baseline characteristics of the study population according to tertiles of METS-VF and METS-IR.

	Total	1.870 ≤ METS-VF < 6.138	6.138 ≤ METS-VF < 6.656	6.656 ≤ METS-VF < 7.932	Total	18.312 ≤ METS-IR < 32.137	32.137 ≤ METS-IR < 37.466	37.466 ≤ METS-IR < 76.940	*p*
		T1	T2	T3		T1	T2	T3	
N	70,513	23,271	23,237	24,005	70,583	23,294	23,292	23,997	
Female (n, %)	45,211 (64.12)	19,378 (83.27)	15,078 (64.89)	10,755 (44.80)	45,247 (64.10)	17,983 (77.20)	14,906 (64.00)	12,358 (51.50)	<0.001
Age (mean ± sd)	54.14 ± 8.32	50.15 ± 7.35	54.00 ± 7.81	58.15 ± 7.77	54.14 ± 8.32	52.38 ± 8.28	54.72 ± 8.14	55.29 ± 8.25	<0.001
Lifestyle									
Alcohol intake (g/week)	124.45 ± 235.37	78.08 ± 148.20	123.36 ± 199.04	162.02 ± 303.75	124.47 ± 235.33	96.04 ± 175.57	120.96 ± 256.06	152.31 ± 256.85	<0.001
Current drinkers (n, %):	31,161 (44.37)	9318 (40.18)	10,121 (43.75)	11,722 (49.02)	31,200 (44.38)	9789 (42.19)	10,215 (44.05)	11,196 (46.84)	<0.001
Current smokers (n, %):	8524 (12.13)	1817 (7.84)	2872 (12.40)	3835 (16.03)	8535 (12.14)	2042 (8.80)	2590 (11.16)	3903 (16.33)	<0.001
Regular exercise (Yes) (n, %):	35,434 (50.40)	12,107 (52.17)	11,935 (51.53)	11,392 (47.57)	35,464 (50.39)	11,950 (51.45)	12,102 (52.12)	11,412 (47.68)	<0.001
Disease									
Hypertension (n, %)	21,364 (30.34)	3393 (14.60)	6631 (28.57)	11,340 (47.29)	21,387 (30.34)	3903 (16.79)	6968 (29.94)	10,516 (43.87)	<0.001
Diabetes (n, %)	7037 (17.19)	837 (6.04)	2045 (15.36)	4155 (30.16)	7044 (17.19)	764 (5.62)	1929 (14.79)	4351 (30.33)	<0.001
Obesity (n, %)	24,120 (34.21)	821 (3.53)	6429 (27.67)	16,870 (70.28)	24,148 (34.21)	164 (0.70)	5530 (23.74)	18,454 (76.90)	<0.001
Abdominal obesity (n, %)	18,065 (25.62)	13 (0.06)	2395 (10.31)	15,657 (65.22)	18,065 (25.62)	695 (2.99)	4147 (17.82)	13,223 (55.16)	<0.001
Hypo-HDL cholesterolemia (n, %)	23,091 (32.75)	5205 (22.37)	7817 (33.64)	10,069 (41.95)	23,107 (32.74)	2291 (9.84)	7057 (30.30)	13,759 (57.34)	<0.001
Hypertriglyceridemia (n, %)	9713 (13.77)	1059 (4.55)	2948 (12.69)	5706 (23.77)	9728 (13.78)	279 (1.20)	1774 (7.62)	7675 (31.98)	<0.001
Hypercholesterolemia (n, %)	8428 (11.95)	2162 (9.29)	3077 (13.24)	3189 (13.28)	8433 (11.95)	2326 (9.99)	2890 (12.41)	3217 (13.41)	<0.001
Dyslipidemia (n, %)	35,263 (50.04)	8070 (34.71)	12,090 (52.06)	15,103 (62.96)	35,294 (50.04)	5687 (24.44)	11,337 (48.70)	18,270 (76.16)	<0.001
Anthropometric traits									
Body mass index (kg/m^2^)	24.03 ± 2.93	21.61 ± 1.88	23.94 ± 1.91	26.46 ± 2.57	24.03 ± 2.93	21.32 ± 1.65	23.93 ± 1.52	26.75 ± 2.40	<0.001
Waist circumference (cm)	81.32 ± 8.72	72.30 ± 4.72	81.30 ± 3.86	90.08 ± 5.63	81.32 ± 8.72	74.05 ± 6.32	81.28 ± 6.00	88.41 ± 6.97	<0.001
Systolic blood pressure (mmHg)	122.23 ± 15.44	116.63 ± 14.33	122.51 ± 14.77	127.40 ± 15.27	122.24 ± 15.44	117.49 ± 14.61	122.60 ± 14.92	126.49 ± 15.43	<0.001
Diastolic blood pressure (mmHg)	75.98 ± 10.03	72.65 ± 9.54	76.21 ± 9.67	78.99 ± 9.83	75.98 ± 10.03	72.96 ± 9.55	76.13 ± 9.67	78.78 ± 9.99	<0.001
Biochemical traits									
Glucose (mg/dL)	95.21 ± 19.93	90.01 ± 13.85	94.77 ± 19.14	100.68 ± 23.87	95.22 ± 19.95	89.33 ± 11.83	94.08 ± 16.75	102.03 ± 26.10	<0.001
HbA1C (%)	5.71 ± 0.74	5.51 ± 0.52	5.71 ± 0.70	5.95 ± 0.90	5.71 ± 0.74	5.51 ± 0.45	5.68 ± 0.66	5.97 ± 0.95	<0.001
Fasting insulin (μU/mL)	7.82 ± 4.42	6.64 ± 3.01	7.27 ± 3.39	8.86 ± 5.40	7.82 ± 4.42	6.26 ± 3.03	7.31 ± 4.57	8.88 ± 4.59	<0.001
TC (mg/dL)	197.51 ± 35.64	194.03 ± 33.62	199.60 ± 35.89	198.86 ± 37.02	197.50 ± 35.64	195.53 ± 33.78	198.22 ± 35.84	198.72 ± 37.09	<0.001
HDL-C (mg/dL)	52.48 ± 13.12	58.44 ± 13.59	52.00 ± 12.12	47.16 ± 11.03	52.48 ± 13.12	62.49 ± 12.63	51.71 ± 9.90	43.51 ± 8.84	<0.001
TG (mg/dL)	129.57 ± 89.02	96.56 ± 57.59	128.64 ± 81.97	162.47 ± 106.87	129.58 ± 89.04	83.03 ± 36.77	118.07 ± 55.13	185.94 ± 116.81	<0.001
γ-GTP (IU/L)	30.67 ± 41.13	21.42 ± 26.43	30.60 ± 41.06	39.76 ± 49.99	30.68 ± 41.15	23.18 ± 33.52	29.32 ± 41.11	39.23 ± 46.01	<0.001
AST (IU/L)	24.20 ± 22.24	22.08 ± 10.86	24.21 ± 32.67	26.24 ± 17.23	24.20 ± 22.24	22.75 ± 13.03	23.68 ± 17.03	26.11 ± 31.65	<0.001
ALT (IU/L)	22.80 ± 22.59	18.11 ± 14.21	22.75 ± 28.83	27.38 ± 21.35	22.80 ± 22.59	18.38 ± 14.96	21.86 ± 19.07	27.99 ± 29.71	<0.001
Clinical Index									
METS-VF	6.33 ± 0.62	5.62 ± 0.44	6.41 ± 0.15	6.95 ± 0.21	6.33 ± 0.62	5.77 ± 0.57	6.39 ± 0.39	6.82 ± 0.35	<0.001
METS-IR	35.34 ± 6.25	30.09 ± 3.79	35.10 ± 4.11	40.66 ± 5.41	35.34 ± 6.25	28.81 ± 2.41	34.74 ± 1.53	42.26 ± 4.15	<0.001

Note: Hypertension was defined as systolic blood pressure ≥ 140 mmHg, diastolic blood pressure ≥ 90 mmHg, a self-reported history of hypertension, or antihypertensive medication use; obesity as BMI ≥ 2 5 kg/m^2^; abdominal obesity as waist circumference ≥ 90 cm in men or ≥85 cm in women; hypo-HDL cholesterolemia as HDL-C < 40 mg/dL in men or <50 mg/dL in women; hypertriglyceridemia as TG ≥ 200 mg/dL; and hypercholesterolemia as total cholesterol ≥ 240 mg/dL. T2DM and dyslipidemia were defined as described in Section 4.2.4. Disease classifications were based on clinical measurements, laboratory data, and self-reported medical histories or medication use, as applicable. The participant numbers represent the initial metabolic tertile populations before the exclusion of individuals with missing diabetes status. Diabetes prevalence percentages were calculated using the final case–control samples with ascertainable diabetes status, as shown in Figure 1.

**Figure 1:**
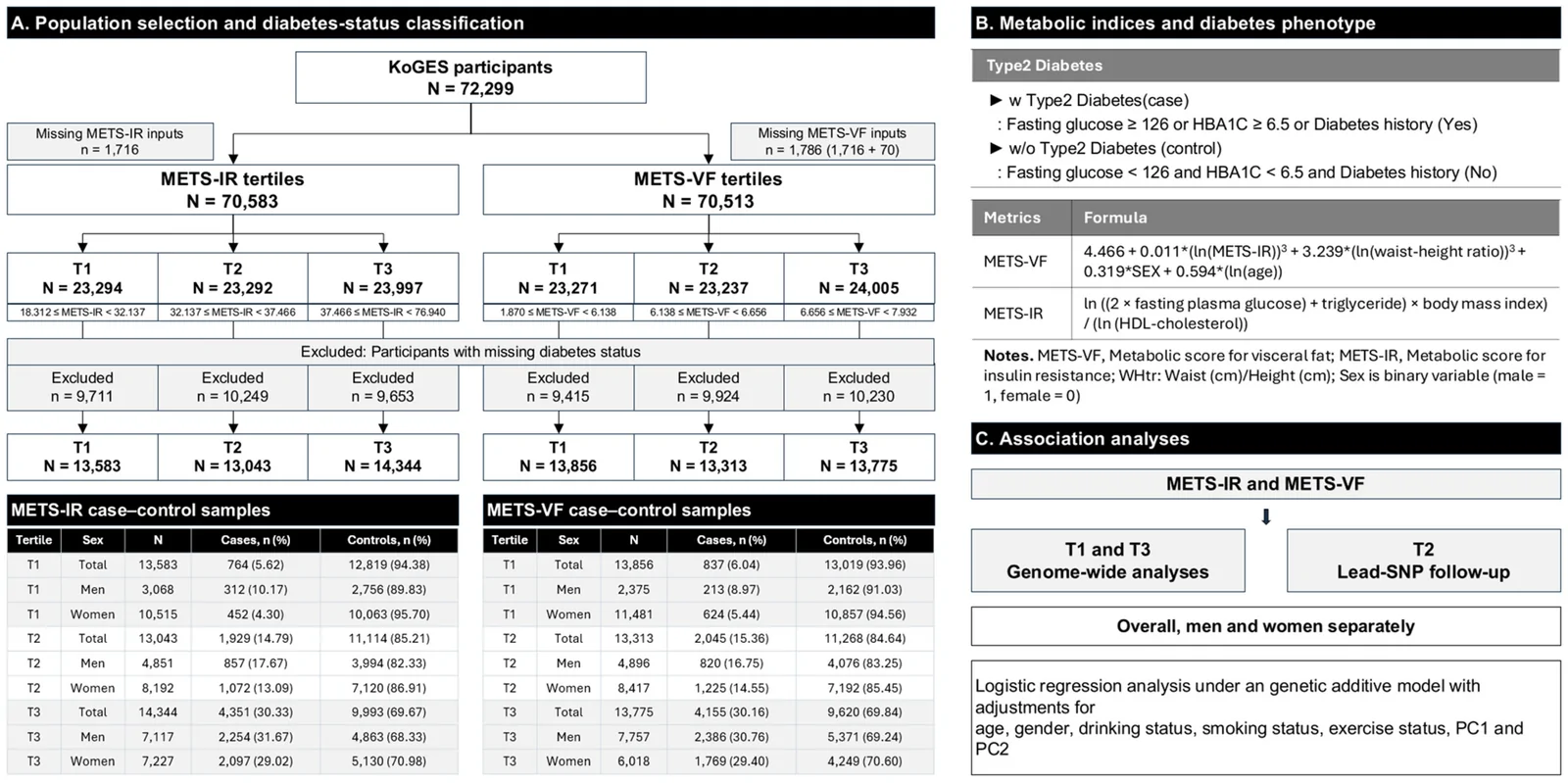
Flowchart of the study population selection and genome-wide association study design for type 2 diabetes according to METS-IR and METS-VF strata.

In both stratification frameworks, the T3 group exhibited a profoundly higher burden of prevalent T2DM than that of the T1 group. Specifically, T2DM prevalence markedly increased from 6.04% in METS-VF T1 to 30.16% in T3, and similarly from 5.62% in METS-IR T1 to 30.33% in T3. Compared with those in the metabolically favorable T1 group, participants in the adverse T3 group had significantly elevated BMI, waist circumference, glucose, HbA1c, fasting insulin, TG levels, and systolic/diastolic BP, along with predictably lower HDL-C levels (*p* < 0.001 for all parameters). These substantial clinical gradients demonstrate that the T1 and T3 strata successfully captured the intended metabolically favorable and unfavorable phenotypic contexts for both visceral adiposity and systemic insulin resistance.

### 2.2. Significant Genome-Wide Loci Across METS-VF and METS-IR Strata

Genome-wide association analyses were performed for prevalent T2DM within each metabolic stratum as defined by METS-VF and METS-IR. Variants with *P* < 5 × 10^−8^ were considered significant genome-wide. Across all sex-stratified analyses, significant genome-wide associations were identified at loci mapped to or near *CDKAL1*, *PAX4*, *DNAJC5B–TRIM55*, *SLC30A8*, *CDKN2A/B*, *CPEB3*, *HHEX*, *CNNM2*, *KCNQ1*, *ALDH2*, *HNF1B*, and *HNF4A*. Genomic annotations and minor allele frequencies of the lead SNPs across populations are summarized in Supplementary Table S2.

Several loci showed genome-wide significant associations across multiple metabolic strata, whereas others reached genome-wide significance only in specific METS-VF, METS-IR, or sex-stratified subgroups. To further evaluate whether genetic effects differed across metabolic environments, lead SNPs identified in the T1 and T3 GWASs were additionally examined in T2, and formal genotype × metabolic stratum interaction and heterogeneity analyses were performed. The stratum-specific association results across T1, T2, and T3, together with interaction *p*-values and heterogeneity statistics, are presented in Table 2. Stratum-specific allele frequencies for the lead SNPs under both METS-VF and METS-IR stratifications are provided in Supplementary Table S3. 

The genomic inflation factors (λGC) ranged from 1.0005 to 1.0450 across the 12 GWAS analyses, indicating minimal overall inflation of the association statistics. The corresponding Q–Q plots are presented in Supplementary Figures S1A–F and S2A–F.

**Figure 2:**
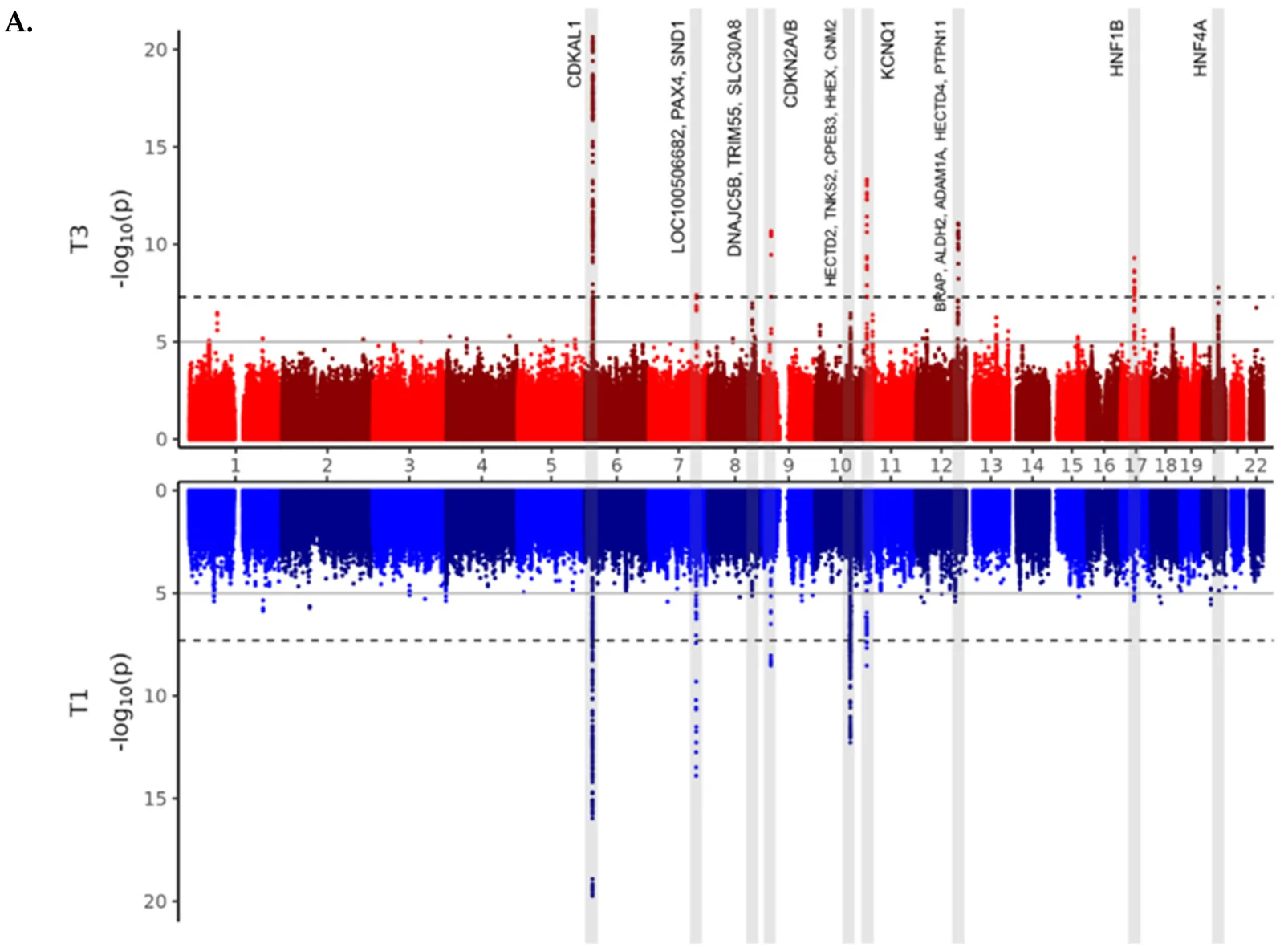

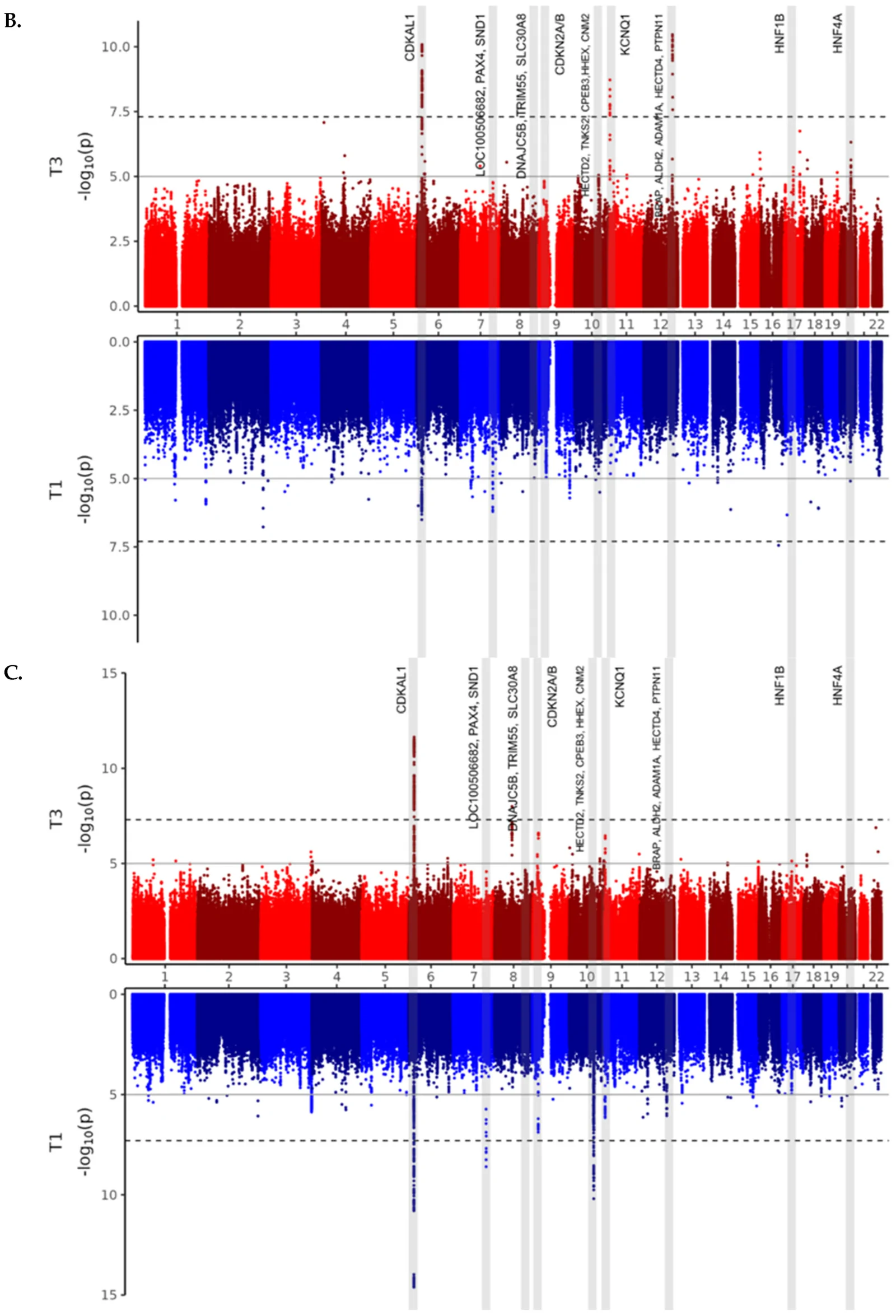
Miami plots of genome-wide associations with type 2 diabetes in the highest and lowest METS-VF tertiles. Miami plots show genome-wide associations with prevalent type 2 diabetes in (**A**) all participants, (**B**) men, and (**C**) women. Each point represents a single-nucleotide polymorphism (SNP), with genomic position on the *x*-axis and −log_10_(P) on the *y*-axis. The upper panels (red) display results from comparisons between type 2 diabetes cases and nondiabetic controls within the highest METS-VF tertile (T3), whereas the lower panels (blue) display the corresponding results within the lowest tertile (T1). Horizontal dashed and solid lines indicate the genome-wide significance threshold (P = 5 × 10^−8^) and the suggestive significance threshold (P = 1 × 10^−5^), respectively.

**Figure 3:**
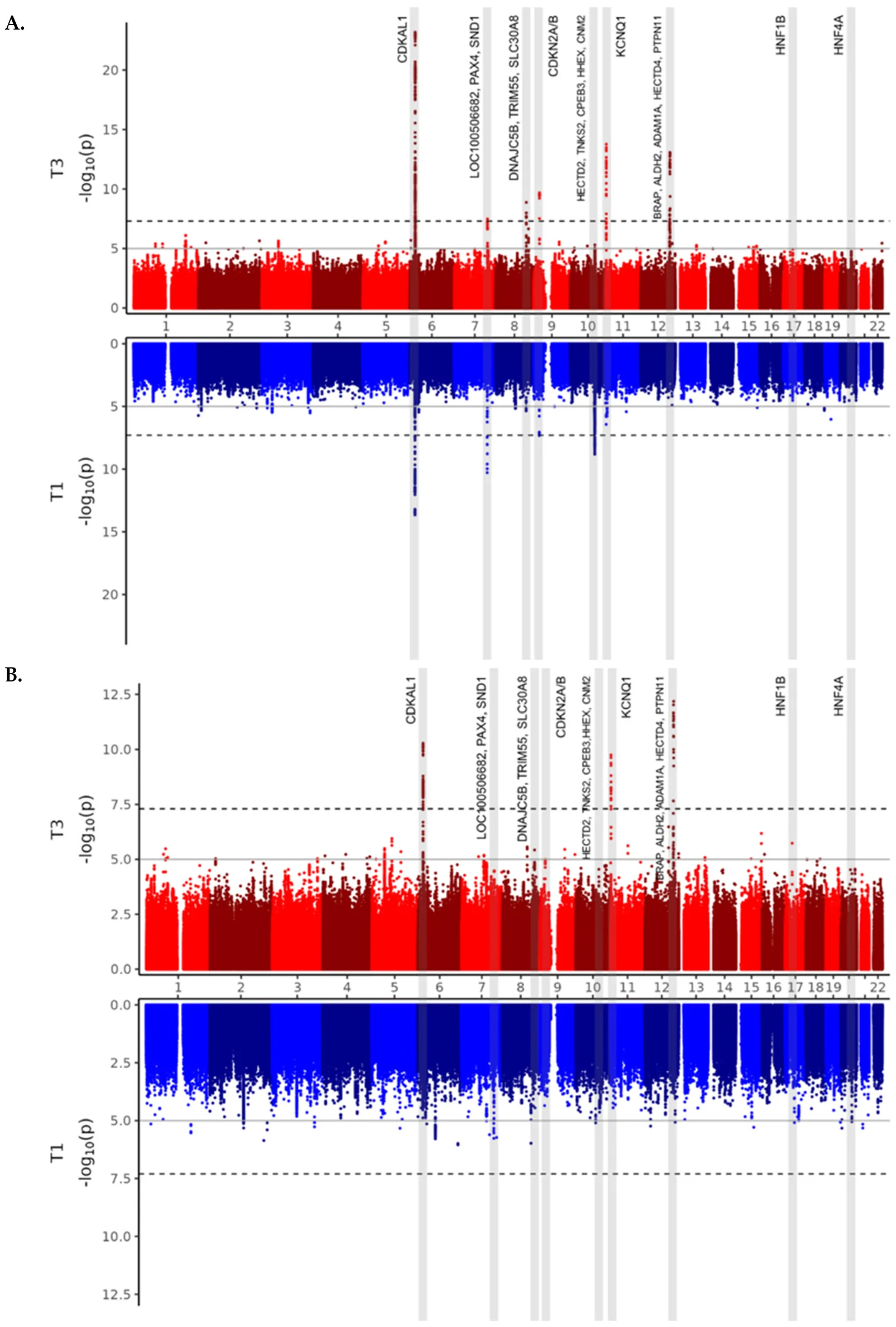

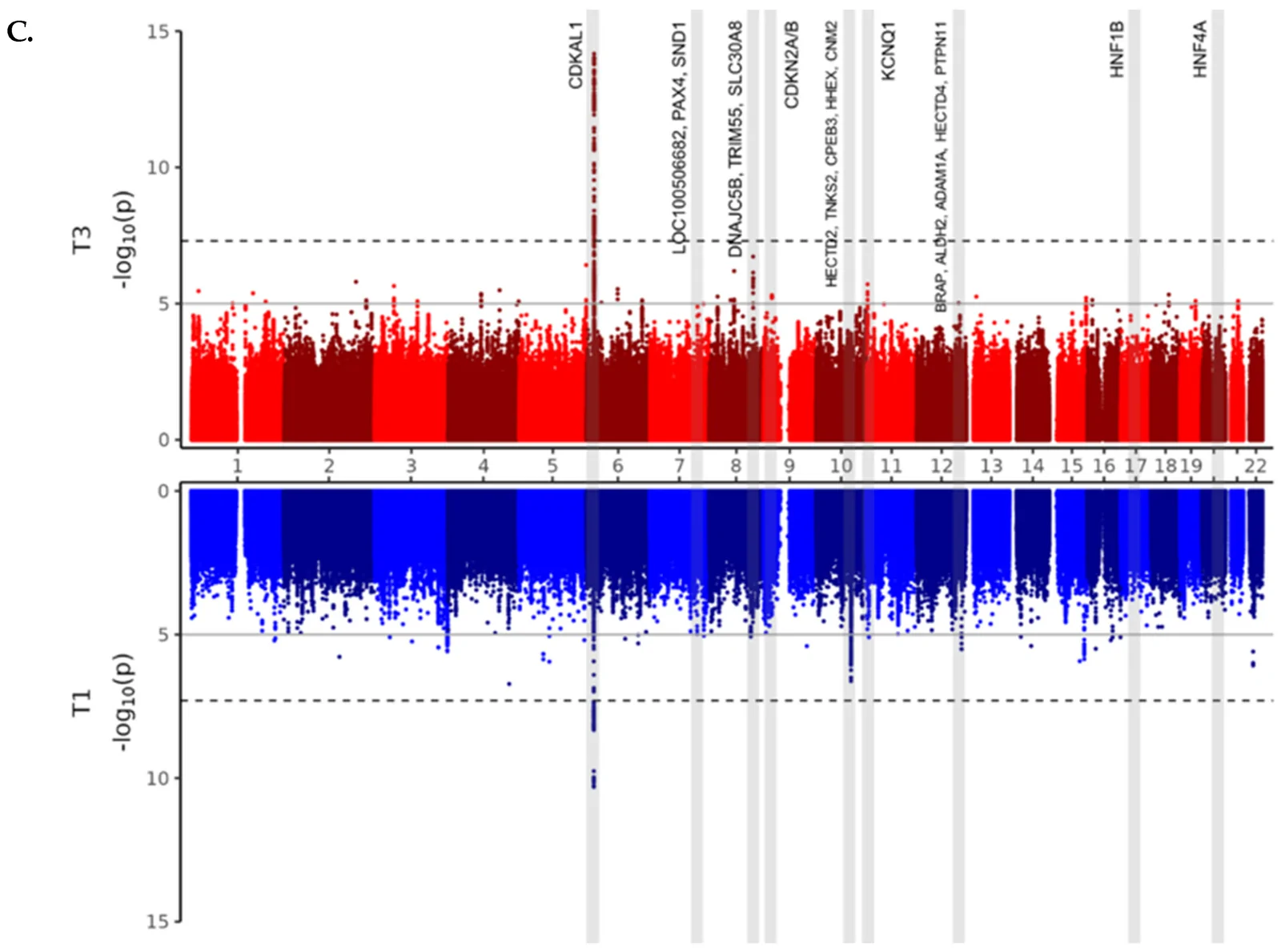
Miami plots of genome-wide associations with type 2 diabetes in the highest and lowest METS-IR tertiles. Miami plots show genome-wide associations with prevalent type 2 diabetes in (**A**) all participants, (**B**) men, and (**C**) women. Each point represents a single-nucleotide polymorphism (SNP), with genomic position on the *x*-axis and −log_10_(P) on the *y*-axis. The upper panels (red) display results from comparisons between type 2 diabetes cases and nondiabetic controls within the highest METS-IR tertile (T3), whereas the lower panels (blue) display the corresponding results within the lowest tertile (T1). Horizontal dashed and solid lines indicate the genome-wide significance threshold (P = 5 × 10^−8^) and the suggestive significance threshold (P = 1 × 10^−5^), respectively.

To facilitate visualization and comparison of genome-wide association signals across metabolic strata, Miami plots were generated for METS-VF (Figure 2) and METS-IR (Figure 3) stratifications. These plots display the Manhattan plots of the T1 and T3 groups in opposing orientations, highlighting both shared and stratum-specific association signals. In addition, regional association plots for five selected T2DM-associated loci (CDKAL1, PAX4, CDKN2A/B, HHEX, and CPEB3) are provided in Supplementary Figure S3 to enable a more detailed examination of the genomic regions surrounding the identified signals.

### 2.3. Loci Consistently Associated with T2DM Across Metabolic Contexts

*CDKAL1* rs9368219 exhibited the most consistent association pattern, reaching genome-wide significance across all four primary metabolic strata in the total population. The risk-increasing T allele showed progressively decreasing effect sizes from T1 to T2 and T3 under both METS-VF (ORs: 1.62, 1.40, and 1.28, respectively) and METS-IR (ORs: 1.52, 1.44, and 1.30, respectively) stratifications. Formal interaction analyses supported differences in genetic effects across metabolic strata (METS-VF: P = 1.55 × 10^−4^; METS-IR: P = 0.0056). Cochran’s Q heterogeneity tests further supported these findings (METS-VF: Q = 15.883, I^2^ = 93.7%, P = 6.74 × 10^−5^; METS-IR: Q = 6.523, I^2^ = 84.7%, P = 0.011). In sex-stratified analyses, the direction of effect attenuation was generally similar, although the statistical evidence for interaction and heterogeneity varied between men and women (Table 2).

*KCNQ1* rs163184 (G allele) also demonstrated consistent risk associations across multiple strata. These included METS-VF T1 (OR 1.36, 95% CI 1.23–1.51, P = 2.99 × 10^−9^), METS-VF T3 (OR 1.18, 95% CI 1.12–1.25, P = 4.33 × 10^−10^), and METS-IR T3 (OR 1.21, 95% CI 1.15–1.27, P = 1.89 × 10^−12^) in the total population. The METS-IR T3 association also reached genome-wide significance in men (OR 1.24, 95% CI 1.15–1.34, P = 1.04 × 10^−8^). Formal interaction analyses revealed a nominally significant difference in genetic effects across METS-VF strata (P = 0.0450), supported by the heterogeneity test (Q = 5.70, I^2^ = 82.5%, P = 0.017). In contrast, neither the interaction (P = 0.904) nor the heterogeneity test (P = 0.764) was significant under METS-IR stratification, suggesting that evidence of metabolic effect modification differed according to the metabolic index used (Table 2).

Conversely, *CDKN2A/B* rs10811662 displayed protective associations across both favorable and unfavorable metabolic strata. The coded A allele was associated with a lower prevalence of T2DM in METS-VF T1 (OR 0.73, 95% CI 0.66–0.81, P = 3.99 × 10^−9^), METS-VF T3 (OR 0.83, 95% CI 0.79–0.88, P = 2.07 × 10^−11^), and METS-IR T3 (OR 0.84, 95% CI 0.80–0.89, P = 2.08 × 10^−10^). Although inverse associations were observed across multiple strata, formal interaction analyses indicated nominally significant differences in genetic effects under both METS-VF and METS-IR stratifications (Table 2). These findings suggest that the protective association of CDKN2A/B was detectable across metabolic environments, although its magnitude may vary according to metabolic context.

### 2.4. Predominant Significant Genome-Wide Loci in Metabolically Favorable Groups

Several loci exhibited significant genome-wide associations, predominantly within the metabolically favorable (T1) groups.

In particular, *PAX4* rs2233580, an East Asian-enriched missense variant, showed stronger associations with T2DM in metabolically favorable strata. In the total population, the odds ratios progressively decreased from T1 to T2 and T3 under both METS-VF (ORs: 1.86, 1.37, and 1.29, respectively) and METS-IR (ORs: 1.75, 1.47, and 1.30, respectively) stratifications. Formal interaction analyses supported differences in genetic effects across metabolic strata (METS-VF: P = 5.21 × 10^−4^; METS-IR: P = 0.0023). Cochran’s Q heterogeneity tests also indicated significant heterogeneity between T1 and T3 (METS-VF: Q = 14.891, I^2^ = 93.3%, P = 1.14 × 10^−4^; METS-IR: Q = 9.055, I^2^ = 89.0%, P = 0.003). In sex-stratified analyses, significant interactions were observed in men under both METS-VF and METS-IR stratifications, whereas in women, a significant interaction was observed only under METS-VF stratification (Table 2).

*HHEX* rs191684511 (G allele) showed stronger associations with T2DM in metabolically favorable strata. In the total population, the odds ratios were 2.21, 1.41, and 1.44 across METS-VF T1, T2, and T3, respectively, and 2.00, 1.65, and 1.36 across the corresponding METS-IR strata. Formal interaction analyses indicated significant differences in genetic effects across metabolic strata (METS-VF: P = 0.0103; METS-IR: P = 0.0079). Cochran’s Q heterogeneity tests also demonstrated significant heterogeneity between T1 and T3 (METS-VF: Q = 10.53, I^2^ = 90.5%, P = 0.001; METS-IR: Q = 7.448, I^2^ = 86.6%, P = 0.006). In sex-stratified analyses, a nominally significant interaction was observed in women under METS-VF stratification (P = 0.0225), whereas the interaction tests in men did not reach statistical significance (Table 2).

*CPEB3* rs183572120 showed stronger associations with T2DM in metabolically favorable strata. In the total population, the odds ratios progressively decreased from T1 to T2 and T3 under both METS-VF (ORs: 1.84, 1.33, and 1.28, respectively) and METS-IR (ORs: 1.70, 1.45, and 1.25, respectively) stratifications. Formal interaction analyses indicated nominally significant differences in genetic effects across metabolic strata (METS-VF: P = 0.0143; METS-IR: P = 0.0188). Cochran’s Q heterogeneity tests also indicated significant heterogeneity between T1 and T3 (METS-VF: Q = 8.373, I^2^ = 88.1%, P = 0.004; METS-IR: Q = 5.576, I^2^ = 82.1%, P = 0.018). In sex-stratified analyses, nominally significant interactions were observed in men under both METS-VF and METS-IR stratifications, whereas neither interaction reached statistical significance in women (Table 2).

In contrast, *CNNM2* rs747219957 showed inverse associations with T2DM across metabolic strata, with stronger associations observed in the metabolically favorable METS-VF T1 group. The variant reached genome-wide significance in both the total population (OR 0.82, 95% CI 0.77–0.87, P = 9.85 × 10^−10^) and women (OR 0.81, 95% CI 0.76–0.87, P = 1.08 × 10^−8^) within METS-VF T1. However, differences in genome-wide statistical significance between subgroups should not be interpreted as evidence of differential genetic effects without supporting interaction or heterogeneity tests. Therefore, these findings are presented as descriptive differences in association patterns rather than definitive evidence of metabolic context-specific or sex-specific genetic effects.

### 2.5. Predominant Significant Genome-Wide Loci in Metabolically Unfavorable Groups

Several loci reached genome-wide significance predominantly in metabolically unfavorable (T3) groups, although subgroup-specific significance did not necessarily indicate significant differences in genetic effects across metabolic strata.

Specifically, *HNF4A* rs12625671 (OR 1.16, 95% CI 1.10–1.23, P = 1.61 × 10^−8^) and *HNF1B* rs11658063 (OR 1.20, 95% CI 1.13–1.27, P = 5.07 × 10^−10^) showed genome-wide significant associations in the total METS-VF T3 subgroup, with modest effect sizes. Although these associations reached genome-wide significance only in METS-VF T3, formal interaction analyses did not provide significant evidence of metabolic effect modification in the total population for either locus (*HNF4A*: METS-VF P = 0.0939, METS-IR P = 0.837; *HNF1B*: METS-VF P = 0.594, METS-IR P = 0.210). Notably, *HNF4A* showed a significant interaction in men under METS-VF stratification (P = 4.28 × 10^−4^), suggesting potential heterogeneity within this sex-stratified analysis (Table 2).

The intergenic variant rs1304925012 near *DNAJC5B–TRIM55* exhibited a subgroup-restricted genome-wide significant association. In women within METS-VF T3, the variant showed an OR of 2.24 (95% CI 1.70–2.96, P = 1.01 × 10^−8^), whereas genome-wide significance was not observed in the total population or in men. In women, formal interaction analysis revealed a nominally significant difference in genetic effects across METS-VF strata (P = 0.0101), supported by the heterogeneity test (P = 0.040). However, this finding indicates potential metabolic effect modification within women and does not establish a female-specific genetic effect without a formal genotype × sex interaction test (Table 2).

*SLC30A8* rs3802177 reached genome-wide significance in the METS-IR T3 group (OR 0.86, 95% CI 0.81–0.90, P = 1.03 × 10^−8^). Although inverse associations were also observed in other metabolic strata, only the METS-IR T3 subgroup met the genome-wide significance threshold. This finding is therefore interpreted as a difference in statistical significance across subgroups rather than definitive evidence of metabolic effect modification.

*ALDH2* rs671 showed genome-wide significant inverse associations in metabolically unfavorable groups, including METS-VF T3 (OR 0.75, 95% CI 0.69–0.82, P = 8.34 × 10^−12^) and METS-IR T3 (OR 0.74, 95% CI 0.69–0.80, P = 1.86 × 10^−13^) in the total population. In men, inverse associations were observed in both METS-VF T3 (OR 0.68, 95% CI 0.61–0.76, P = 3.41 × 10^−11^) and METS-IR T3 (OR 0.65, 95% CI 0.57–0.73, P = 9.50 × 10^−13^). Formal interaction analyses demonstrated significant differences in genetic effects across metabolic strata in the total population under both METS-VF (P = 3.69 × 10^−4^) and METS-IR (P = 0.00164) stratifications. Although the inverse associations appeared more pronounced in men, these sex-stratified findings do not establish sex-specific genetic effects without a formal genotype × sex interaction test (Table 2).

## 3. Discussion

In this study, the genetic architecture of T2DM was investigated according to metabolic contexts defined by two surrogate indices (METS-IR and METS-VF), using genome-wide genotype data from approximately 70,000 Korean individuals. By conducting GWASs within metabolically favorable and unfavorable strata, 12 genome-wide significant loci were identified. Additional genotype × metabolic stratum interaction and heterogeneity analyses provided evidence of variation in genetic effects across metabolic environments for selected loci, particularly *CDKAL1* and *PAX4*. Other loci exhibited differences in genome-wide statistical significance across metabolic or sex-stratified subgroups without uniformly significant interaction effects. These findings suggest that metabolic stratification can reveal both shared genetic associations and potential effect modification, while emphasizing the importance of distinguishing subgroup-specific statistical significance from genuine differences in genetic effects.

The associations observed for *CDKAL1* [16], *CDKN2A/B* [17], and *KCNQ1* [18] support the central role of β-cell dysfunction and impaired insulin secretion in T2DM susceptibility. Although these loci were associated with T2DM across multiple metabolic strata, their effect sizes were not necessarily uniform across metabolic environments. Notably, *CDKAL1* demonstrated significant genotype × metabolic stratum interactions under both METS-VF and METS-IR stratifications, with progressively attenuated effect estimates from T1 to T3. *CDKN2A/B* also showed nominally significant interactions under both indices, whereas *KCNQ1* showed evidence of interaction under METS-VF but not METS-IR stratification. *CDKAL1* has been strongly implicated in pancreatic β-cell function and insulin secretion [19], while *CDKN2A/B* [17] and *KCNQ1* [13] are established T2DM susceptibility loci, particularly in East Asian populations. Together, these findings indicate that established β-cell-related genetic associations remain detectable across metabolic environments, although their magnitudes may vary according to metabolic context.

Importantly, some loci showed stronger associations in metabolically favorable groups. In particular, *PAX4*, *CPEB3*, and *HHEX* exhibited larger effect estimates in T1 than in T3 under both METS-IR and METS-VF stratifications, with nominally significant interaction and heterogeneity tests supporting differences in genetic effects across metabolic strata. These findings suggest that β-cell-related genetic susceptibility may be more apparent when metabolic burden is relatively limited. In individuals with low insulin resistance or low visceral adiposity, inherited defects in insulin secretion, pancreatic development, or glucose sensing may contribute to T2DM susceptibility. However, these observations do not establish the underlying biological mechanisms, and further functional studies are needed to determine whether metabolic conditions directly modify the effects of these variants.

The detection of *CPEB3* and *CNNM2* in specific metabolic or sex-stratified subgroups may provide additional insights into less well-characterized genetic associations with T2DM. Notably, *CPEB3* has not been studied as extensively as classical T2DM loci, and its larger effect estimates in metabolically favorable groups, supported by nominally significant interaction tests, suggest a potentially underrecognized association pattern in Korean or East Asian populations. *CNNM2* reached genome-wide significance in the total population and in women within the METS-VF T1 subgroup. However, formal interaction analyses did not demonstrate significant differences in genetic effects across metabolic strata in the total population. Therefore, the *CNNM2* findings should be interpreted as subgroup-dependent differences in statistical significance rather than definitive evidence of metabolic context-specific or sex-specific genetic effects. Independent replication is warranted.

In contrast, *HNF1B* and *HNF4A* reached genome-wide significance predominantly in the metabolically unfavorable METS-VF T3 group. Both genes are involved in the transcriptional regulation of glucose metabolism and pancreatic β-cell function [20,21]. However, formal interaction analyses in the total population did not demonstrate significant differences in genetic effects across metabolic strata for either locus. Therefore, the T3-predominant genome-wide significance should not be interpreted as definitive evidence that adverse metabolic conditions enhance their genetic effects. Notably, *HNF4A* showed a significant interaction in men under METS-VF stratification, suggesting a potential metabolic effect modification within this subgroup that requires further validation. Visceral adiposity is associated with lipotoxicity, chronic inflammation, and hepatic insulin resistance, which may contribute to T2DM development [9]. Previous genetic evidence has also implicated inflammation-related pathways in T2DM susceptibility [11]. Nevertheless, inflammatory signaling was not directly evaluated in the present study, and the proposed biological connections remain exploratory.

The *SLC30A8* locus reached genome-wide significance in the METS-IR T3 group. *SLC30A8* encodes a zinc transporter involved in insulin granule formation and β-cell function [22]. Although this association was most statistically significant in the high METS-IR stratum, formal interaction analyses did not demonstrate significant differences in genetic effects across metabolic strata. Thus, the observed pattern does not establish a preferential genetic effect under conditions of increased insulin resistance. Previous studies have implicated genes involved in insulin signaling, including *INSR*, *IRS1*, and *AKT2*, in the regulation of insulin sensitivity and T2DM susceptibility. These pathways provide a biological context for understanding genetic susceptibility under conditions of increased insulin resistance. Although these genes were not among the principal loci highlighted in our analyses, their established roles underscore the importance of considering insulin-signaling mechanisms alongside pancreatic β-cell dysfunction when interpreting metabolically stratified GWAS findings [23]. Previous candidate-gene research has also reported an association between INPPL1 polymorphisms and T2DM susceptibility in a North Indian population [24].

A notable subgroup-dependent association was observed for the intergenic variant near *DNAJC5B–TRIM55*. This variant reached genome-wide significance in women within the METS-VF T3 subgroup, with an odds ratio of 2.24. Formal interaction analysis in women also showed a nominally significant genotype × metabolic stratum interaction under METS-VF stratification (*p* = 0.0101), suggesting potential variation in genetic effects across metabolic environments within this subgroup. However, this finding does not establish a sex-specific genetic effect, as a formal genotype × sex interaction was not performed. Differences in fat distribution, hormonal regulation, and metabolic responses to visceral adiposity may provide possible biological explanations [25], although these mechanisms were not directly investigated. Given the low allele frequency and subgroup-specific nature of the signal, independent replication and functional validation are required.

The *ALDH2* locus was inversely associated with T2DM risk, particularly in men in the METS-VF T3 group. Formal interaction analyses further demonstrated significant differences in the association of *ALDH2* with T2DM across metabolic strata in the total population under both METS-VF (P = 3.69 × 10^−4^) and METS-IR (P = 0.00164) stratifications. These findings suggest potential metabolic effect modification, although the underlying contribution of alcohol-related behavioral pathways remains uncertain. *ALDH2* is a well-known East Asian-enriched gene involved in alcohol metabolism [26]. Individuals carrying reduced-function *ALDH2* variants often experience acetaldehyde-related flushing and tend to consume less alcohol [27]. Therefore, the observed inverse association may partly reflect behavioral modifications related to alcohol intolerance, rather than a direct protective biological effect on glucose metabolism. This finding highlights the importance of considering gene–environment relationships when interpreting genetic associations, particularly for variants with strong behavioral consequences. Importantly, alcohol consumption was included as a covariate in our GWAS models, although drinking behavior may itself be influenced by *ALDH2* genotype. Therefore, adjustment for alcohol consumption may partially account for a pathway through which *ALDH2* variants are associated with T2DM risk, potentially affecting the estimated genetic association. Residual confounding related to alcohol consumption patterns and other behavioral factors may also remain; thus, the observed inverse association should not be interpreted as evidence of a direct protective effect of *ALDH2* variants.

A comparison between METS-IR and METS-VF stratification provides important methodological implications. Specifically, METS-VF-based analyses produced clearer and more distinct association peaks than those of METS-IR-based analyses. This may be because visceral adiposity represents a more direct and stable metabolic stressor that contributes to T2DM through lipotoxicity, inflammation, and β-cell stress [9]. In contrast, METS-IR may reflect a more heterogeneous and dynamic metabolic state influenced by glycemia, adiposity, lifestyle, medication use, and disease progression [28]. Therefore, stratification by visceral adiposity may improve phenotypic contrast and enhance the detectability of genetic effects in T2DM GWASs. Previous studies have highlighted the interplay between genetic susceptibility to T2DM and metabolic factors, including obesity, lipoprotein profiles, and nutritional status [29]. These findings provide a broader context for our metabolically stratified GWAS approach, suggesting that genetic associations with T2DM may vary according to the underlying metabolic environment. Previous candidate-gene studies have investigated the associations of adiposity- and insulin resistance-related genetic variants with T2DM susceptibility, including polymorphisms in genes encoding resistin and urotensin II [30]. These findings further support the relevance of considering metabolic characteristics when interpreting genetic susceptibility to T2DM. Several loci, including HNF1B, HNF4A, and DNAJC5B–TRIM55, reached genome-wide significance predominantly in METS-VF-defined subgroups. However, these differences in statistical significance do not necessarily indicate stronger genetic effects under METS-VF stratification. The distinct association patterns observed between the two metabolic indices may reflect differences in subgroup characteristics, statistical power, or the metabolic dimensions captured by each index. Further studies are needed to determine whether visceral adiposity and insulin resistance differentially modify genetic susceptibility to T2DM.

This study has several strengths. In particular, it analyzed a large, well-characterized Korean cohort with genome-wide genotypic data and detailed clinical and lifestyle information. By applying both METS-IR and METS-VF, the study compared two biologically related but distinct metabolic contexts. In addition, sex-stratified analyses enabled an exploration of genetic association patterns within men and women.

However, this study has some limitations. First, it was based on prevalent T2DM rather than incident disease, which limits causal interpretation. Second, METS-IR and METS-VF are surrogate indices rather than direct measures of insulin resistance or visceral fat. Furthermore, METS-IR incorporates fasting glucose, and METS-VF includes METS-IR as a component, whereas fasting glucose is also used to define T2DM. This overlap may contribute to differences in T2DM prevalence across metabolic strata and potentially influence the observed genetic association patterns. Therefore, these findings should be interpreted with caution, and future studies using metabolic stratification measures independent of glycemic parameters are warranted. Third, although stratification by the extreme tertiles of METS-IR and METS-VF was intended to increase the contrast between metabolic environments and facilitate the exploration of potentially context-specific genetic associations, this approach reduced the sample size within each stratum compared with an analysis of the full cohort, thereby limiting statistical power, particularly for low-frequency variants. Consequently, the absence of genome-wide significance in one stratum should not be interpreted as evidence of the absence of a genetic effect, and differences in statistical significance between strata do not necessarily indicate genuine effect heterogeneity. Although formal interaction and heterogeneity analyses were performed for selected lead variants, multiple comparisons across loci, metabolic indices, and sex-stratified subgroups increase the possibility of chance findings. Accordingly, nominally significant interaction results should be interpreted cautiously and require validation in independent cohorts. Furthermore, sex-stratified interaction analyses assess metabolic effect modification within each sex but do not directly establish differences in genetic effects between men and women, as formal genotype × sex interaction tests were not performed. Therefore, the observed subgroup-dependent association patterns should be considered exploratory until independently replicated.

## 4. Materials and Methods

### 4.1. Study Population

The study population was derived from KoGES, which is a large-scale population-based cohort. The cohort was established to investigate the genetic, environmental, and lifestyle determinants of complex diseases, including T2DM, in the Korean population. KoGES provides comprehensive epidemiological, clinical, biochemical, lifestyle, and genome-wide genotype information [31].

Initially, genome-wide single-nucleotide polymorphism (SNP) genotype data were available for 72,299 participants. After excluding 1716 individuals with missing clinical or biochemical variables required for METS-IR calculation, 70,583 participants were eligible for METS-IR-based stratification. Subsequently, an additional 70 individuals with missing variables required for METS-VF calculation were excluded, resulting in 70,513 participants eligible for METS-VF-based stratification.

Participants were first categorized into tertiles separately for each metabolic index, before excluding those with missing diabetes status. The METS-IR tertiles comprised 23,294 participants in T1, 23,292 in T2, and 23,997 in T3. After excluding 9711, 10,249, and 9653 participants with missing diabetes status from the respective tertiles, the final METS-IR case–control sample included 40,970 participants: 13,583 in T1, 13,043 in T2, and 14,344 in T3. The METS-VF tertiles initially comprised 23,271 participants in T1, 23,237 in T2, and 24,005 in T3. After excluding 9415, 9924, and 10,230 participants with missing diabetes status, respectively, the final METS-VF case–control sample included 40,944 participants: 13,856 in T1, 13,313 in T2, and 13,775 in T3. The original tertile assignments were retained after these exclusions.

The lowest (T1) and highest (T3) tertiles were selected to represent relatively favorable and unfavorable metabolic environments, respectively. This extreme-tertile approach was intended to maximize the contrast between metabolic profiles and facilitate the exploration of potentially context-specific genetic association patterns that might be less apparent in an analysis of the overall cohort. The middle tertile (T2) was excluded from the primary stratified analyses to maintain this metabolic contrast, although this approach reduced the sample size and statistical power available for each GWAS. A schematic overview of the participant selection and study design is presented in Figure 1.

### 4.2. Clinical Measurements and Definition of Type 2 Diabetes

#### 4.2.1. Anthropometric and Clinical Measurements

Trained medical personnel obtained anthropometric measurements using standardized clinical protocols. Body weight and height were measured with the participants wearing light clothing and no shoes. BMI (kg/m^2^) was calculated as weight (kg) divided by height squared (m^2^). Waist circumference was measured to the nearest 0.1 cm at the midpoint between the lower rib margin and iliac crest. Blood pressure (BP) was measured at least twice in a seated position using an automated sphygmomanometer after a sufficient resting period of at least 5 min. The average of the recorded BP measurements was used for subsequent analyses.

#### 4.2.2. Biochemical Analysis

Peripheral blood samples were collected after fasting overnight for at least 8 h. Biochemical parameters, including fasting plasma glucose (FPG), glycated hemoglobin (HbA1c), liver enzyme levels, and lipid profiles (total cholesterol, triglycerides [TG], high-density lipoprotein cholesterol [HDL-C], and low-density lipoprotein cholesterol [LDL-C]) were measured using standardized and quality-controlled laboratory methods.

#### 4.2.3. Lifestyle Covariates

Lifestyle factors were assessed comprehensively using structured questionnaires administered by trained interviewers. Smoking status was categorized as never smoker, former smoker, or current smoker. Alcohol consumption was classified as never, former, or current use, with additional detailed data on drinking frequency and daily intake collected from current drinkers. Regular physical activity was defined as engaging in exercise or moderate-to-vigorous physical activity for at least 30 min/day.

#### 4.2.4. Definitions of T2DM and Dyslipidemia

Prevalent T2DM was defined based on the fulfillment of at least one of the following criteria: FPG ≥ 126 mg/dL, HbA1c ≥ 6.5%, a self-reported history of physician-diagnosed diabetes, or current use of antidiabetic medications. Participants who did not meet any of these criteria were classified as nondiabetic controls. Dyslipidemia was defined as meeting any of the following criteria: total cholesterol ≥ 240 mg/dL, TG ≥ 200 mg/dL, HDL-C < 40 mg/dL in men or <50 mg/dL in women, a self-reported history of dyslipidemia, or current use of lipid-lowering medications (e.g., statins).

### 4.3. Assessment of Metabolic Phenotypes

The validated surrogate indices METS-IR and METS-VF were used to characterize metabolic environments related to systemic insulin resistance and visceral adiposity. METS-IR was calculated using FPG, TG, BMI, and HDL-C according to the following formula:

METS-IR = ln[(2 × FPG) + TG] × BMI/ln(HDL-C)

METS-VF was calculated using the validated equation proposed by Bello-Chavolla et al. [8]:

METS-VF = 4.466 + 0.011 × [ln(METS-IR)]^3^ + 3.239 × [ln(WHtR)]^3^ + 0.319 × sex + 0.594 × ln(age)

where WHtR represents the waist-to-height ratio, age is expressed in years, and sex is coded as a binary variable (male = 1; female = 0).

Genome-wide association analyses for prevalent T2DM were performed separately within the T1 and T3 groups for each index by comparing T2DM cases with non-diabetic controls. Additional sex-stratified GWASs were conducted independently for men and women within each metabolic stratum. The same tertile assignments were used for the overall and sex-stratified analyses. The intermediate tertile (T2) was used for targeted follow-up analyses of lead SNPs identified in the T1 and/or T3 GWASs. The overall study design is shown in Figure 1.

### 4.4. Genotyping and Quality Control

Genomic DNA was extracted from peripheral blood samples using standardized protocols. DNA samples were deposited and managed by the National Biobank of Korea for long-term preservation and research purposes. Genome-wide genotyping was performed using the Korea Biobank Array (KoreanChip), which was specifically designed to capture genetic variants commonly observed in Korean populations. Detailed information on the array design and performance has been previously described by Moon et al. [32]. Genomic coordinates were reported according to the GRCh37 reference genome.

The present study utilized existing genotype data from the Korean Genome and Epidemiology Study (KoGES). A sample-level genetic quality control was performed as part of the original KoGES genotyping and data-processing procedures, and the resulting genotype data were used for the present GWAS analyses. SNP-level quality control procedures were applied to ensure the reliability of the genetic data. SNPs were excluded if they had a low call rate, high absence, deviation from Hardy–Weinberg equilibrium, or low minor allele frequency. Specifically, SNPs with a call rate < 97%, absence >1%, Hardy–Weinberg equilibrium P < 1 × 10^−6^, or minor allele frequency <1% were removed. After quality-control filtering, the remaining SNPs were used for genome-wide association analyses.

### 4.5. Genome-Wide Association Analysis

Genome-wide association analyses were performed to identify genetic variants associated with T2DM within each metabolic phenotype group. Analyses were conducted separately within the lowest (T1) and highest (T3) tertiles of METS-IR and METS-VF. Within each stratum, T2DM cases were compared with non-diabetic controls using logistic regression under an additive genetic model. Additional sex-stratified GWASs were performed separately in men and women within each stratum.

All models were adjusted for age, sex, alcohol intake, smoking status, physical activity, and the first two genetic principal components (PC1 and PC2). Sex-stratified models included the same covariates except sex. All association analyses were conducted using PLINK version 1.9 [33].

Genome-wide significance was defined as P < 5 × 10^−8^. Genome-wide significant variants were grouped into gene-centered regions defined by the annotated gene boundaries and 100-kb flanking intervals on either side. Within each region, the variant with the lowest *p* value was selected as the lead SNP. Lead SNPs identified in the T1 and/or T3 GWASs were subsequently evaluated in the intermediate tertile (T2) using the same additive genetic model and covariate adjustments to compare genetic associations across all three metabolic strata.

To assess whether genetic effects differed across metabolic environments, genotype × metabolic stratum interaction analyses were performed for the major loci identified in the GWASs. In addition, Cochran’s Q heterogeneity tests were conducted to compare genetic effect estimates between the lowest (T1) and highest (T3) tertiles, with heterogeneity quantified using the Q statistic, I^2^, and corresponding *p*-values. These analyses were performed separately for METS-IR and METS-VF in the overall population and in men and women.

### 4.6. Visualization and Locus Annotation

Miami plots were generated using R (version 4.1.2; https://www.r-project.org/; accessed on 11 June 2024) to visualize and compare genome-wide association signals for prevalent T2DM between the T1 and T3 strata of each metabolic index, both in the overall study population and separately in men and women. Quantile–quantile (Q–Q) plots were generated using Python (version 3.9.12) to compare the observed distribution of association *p*-values with the expected null distribution. Genomic inflation factors (λGC) were calculated to assess potential inflation of the GWAS test statistics.

For loci showing genome-wide significant associations, regional association plots were generated using LocusZoom (version 0.4.8.2) to examine local association patterns, linkage disequilibrium structures, and nearby genes. Candidate genes were prioritized based on their physical proximity to the lead SNPs, evidence from previous T2DM GWASs, and their biological relevance to glucose metabolism, insulin secretion, insulin resistance, adiposity, or metabolic stress pathways.

### 4.7. Ethical Approval

All study procedures were conducted in accordance with the ethical principles of the Declaration of Helsinki. Written informed consent was obtained from all participants before enrollment. The study protocol was approved by the Institutional Review Board of Dong-A University Hospital (DAUHIRB-EXP-25-138).

## 5. Conclusions

This study suggests that genetic associations with T2DM may vary across metabolic contexts defined by insulin resistance and visceral adiposity, particularly at selected genetic loci. Although established T2DM susceptibility loci, including *CDKAL1*, *CDKN2A/B*, and *KCNQ1*, showed associations across metabolic strata, formal interaction and heterogeneity analyses provided evidence of variation in genetic effect estimates at selected loci. Differences observed in sex-stratified analyses should be interpreted cautiously, as formal genotype × sex interaction tests were not performed. These findings suggest that metabolic stratification may provide additional insights into the genetic susceptibility to T2DM in the Korean population. Further validation in independent and ethnically diverse cohorts is warranted.

## Figures and Tables

**Table 2:** Associations of lead SNPs with diabetes prevalence across METS-VF and METS-IR tertiles, with interaction and heterogeneity tests. Lead SNPs identified as genome-wide were significantly associated with diabetes prevalence across subgroups.

SNP	Coded Allele	Population	Index	T1		T2		T3		Interaction	Q	I^2^ (%)	Heterogeneity
				OR (95%CI)	*p*-Value	OR (95%CI)	*p*-Value	OR (95%CI)	*p*-Value	*p*-Value			*p*-Value
rs9368219 (CDKAL1)	T	Total	METS-VF	**1.62 (1.46–1.79)**	**1.83 × 10^−20^**	**1.40 (1.31–1.50)**	**1.52 × 10^−22^**	**1.28 (1.22–1.35)**	**1.11 × 10^−20^**	**1.55 × 10^−4^**	15.88	93.70	6.74 × 10^−5^
	T		METS-IR	**1.52 (1.37–1.69)**	**2.20 × 10^−14^**	**1.44 (1.34–1.54)**	**7.79 × 10^−24^**	**1.30 (1.24–1.37)**	**1.35 × 10^−23^**	**5.55 × 10^−3^**	6.52	84.70	1.10 × 10^−2^
	T	Male	METS-VF	1.66 (1.35–2.03)	1.22 × 10^−6^	**1.37 (1.23–1.52)**	**1.38 × 10^−8^**	**1.25 (1.17–1.34)**	**1.52 × 10^−10^**	**9.53 × 10^−3^**	6.50	84.60	1.10 × 10^−2^
	T	Male	METS-IR	1.42 (1.20–1.68)	5.61 × 10^−5^	**1.42 (1.27–1.58)**	**2.16 × 10^−10^**	**1.27 (1.18–1.37)**	**8.48 × 10^−11^**	9.35 × 10^−2^	1.32	24.40	2.50 × 10^−1^
	T	Female	METS-VF	1.61 (1.43–1.81)	**2.49 × 10^−15^**	**1.43 (1.31–1.56)**	**7.52 × 10^−16^**	**1.33 (1.22–1.44)**	**6.96 × 10^−12^**	**1.58 × 10^−2^**	7.05	85.80	8.00 × 10^−3^
	T	Female	METS-IR	1.59 (1.39–1.83)	**4.99 × 10^−11^**	**1.45 (1.32–1.59)**	**5.61 × 10^−15^**	**1.34 (1.24–1.44)**	**1.17 × 10^−14^**	**3.19 × 10^−2^**	4.82	79.30	2.80 × 10^−2^
rs2233580 (PAX4)	T	Total	METS-VF	1.86 (1.59–2.19)	**3.36 × 10^−14^**	1.37 (1.22–1.55)	3.12 × 10^−7^	1.29 (1.17–1.42)	1.49 × 10^−7^	**5.21 × 10^−4^**	14.89	93.30	1.14 × 10^−4^
rs2233580	T	Total	METS-IR	1.75 (1.48–2.08)	**1.06 × 10^−10^**	1.47 (1.30–1.66)	**1.39 × 10^−9^**	1.30 (1.18–1.43)	6.60 × 10^−8^	**2.34 × 10^−3^**	9.06	89.00	3.00 × 10^−3^
rs2233580	T	Male	METS-VF	2.18 (1.60–2.97)	7.12 × 10^−7^	1.46 (1.22–1.76)	4.93 × 10^−5^	1.29 (1.14–1.46)	5.55 × 10^−5^	**3.27 × 10^−3^**	9.60	89.60	2.00 × 10^−3^
rs2233580	T	Male	METS-IR	1.86 (1.44–2.41)	2.89 × 10^−6^	1.53 (1.27–1.84)	7.17 × 10^−6^	1.29 (1.13–1.47)	1.33 × 10^−4^	**6.90 × 10^−3^**	6.06	83.50	1.40 × 10^−2^
rs2233580	T	Female	METS-VF	1.76 (1.46–2.13)	**5.58 × 10^−9^**	1.31 (1.12–1.54)	1.01 × 10^−3^	1.30 (1.12–1.50)	6.24 × 10^−4^	**2.91 × 10^−2^**	6.16	83.80	1.30 × 10^−2^
rs2233580	T	Female	METS-IR	1.65 (1.32–2.08)	1.39 × 10^−5^	1.43 (1.21–1.69)	2.93 × 10^−5^	1.32 (1.15–1.51)	9.67 × 10^−5^	1.24 × 10^−1^	2.82	64.50	9.30 × 10^−2^
rs1304925012 (DNAJC5B-TRIM55)	G	Total	METS-VF	1.11 (0.75–1.64)	6.07 × 10^−1^	1.07 (0.81–1.41)	6.28 × 10^−1^	1.42 (1.16–1.73)	5.77 × 10^−4^	1.33 × 10^−1^	1.22	18.20	2.69 × 10^−1^
rs1304925012	G	Total	METS-IR	1.15 (0.77–1.73)	4.95 × 10^−1^	1.10 (0.83–1.46)	5.17 × 10^−1^	1.41 (1.16–1.73)	7.39 × 10^−4^	1.88 × 10^−1^	0.79	0.00	3.74 × 10^−1^
rs1304925012	G	Male	METS-VF	0.54 (0.19–1.50)	2.34 × 10^−1^	1.21 (0.80–1.83)	3.69 × 10^−1^	0.88 (0.65–1.20)	4.20 × 10^−1^	9.69 × 10^−1^	0.83	0.00	3.62 × 10^−1^
rs1304925012	G	Male	METS-IR	0.99 (0.50–1.96)	9.78 × 10^−1^	0.93 (0.59–1.44)	7.35 × 10^−1^	0.93 (0.68–1.27)	6.59 × 10^−1^	9.29 × 10^−1^	0.03	0.00	8.73 × 10^−1^
rs1304925012	G	Female	METS-VF	1.32 (0.87–2.02)	1.95 × 10^−1^	0.98 (0.68–1.43)	9.35 × 10^−1^	2.24 (1.70–2.96)	**1.01 × 10^−8^**	**1.01 × 10^−2^**	4.22	76.30	4.00 × 10^−2^
rs1304925012	G	Female	METS-IR	1.27 (0.77–2.09)	3.48 × 10^−1^	1.25 (0.87–1.80)	2.35 × 10^−1^	1.98 (1.51–2.58)	6.38 × 10^−7^	5.11 × 10^−2^	2.34	57.30	1.26 × 10^−1^
rs3802177 (SLC30A8)	A	Total	METS-VF	0.80 (0.72–0.89)	3.69 × 10^−5^	0.85 (0.79–0.91)	4.02 × 10^−6^	0.87 (0.82–0.91)	1.05 × 10^−7^	2.64 × 10^−1^	1.61	37.90	2.04 × 10^−1^
rs3802177	A	Total	METS-IR	0.79 (0.71–0.88)	3.00 × 10^−5^	0.85 (0.79–0.92)	2.11 × 10^−5^	0.86 (0.81–0.90)	**1.03 × 10^−8^**	3.07 × 10^−1^	1.69	40.90	1.93 × 10^−1^
rs3802177	A	Male	METS-VF	0.76 (0.61–0.95)	1.40 × 10^−2^	0.90 (0.80–1.00)	5.20 × 10^−2^	0.88 (0.82–0.95)	4.41 × 10^−4^	4.43 × 10^−1^	1.56	35.80	2.12 × 10^−1^
rs3802177	A	Male	METS-IR	0.83 (0.70–0.99)	4.32 × 10^−2^	0.84 (0.75–0.94)	1.88 × 10^−3^	0.88 (0.82–0.95)	6.63 × 10^−4^	4.36 × 10^−1^	0.32	0.00	5.74 × 10^−1^
rs3802177	A	Female	METS-VF	0.81 (0.72–0.92)	7.68 × 10^−4^	0.82 (0.75–0.89)	1.21 × 10^−5^	0.84 (0.78–0.91)	4.26 × 10^−5^	6.50 × 10^−1^	0.21	0.00	6.44 × 10^−1^
rs3802177	A	Female	METS-IR	0.76 (0.66–0.88)	1.83 × 10^−4^	0.86 (0.78–0.95)	2.94 × 10^−3^	0.83 (0.77–0.90)	2.19 × 10^−6^	5.49 × 10^−1^	1.13	11.40	2.88 × 10^−1^
rs10811662 (CDKN2A/B)	A	Total	METS-VF	0.73 (0.66–0.81)	**3.99 × 10^−9^**	0.77 (0.72–0.83)	**3.12 × 10^−13^**	0.83 (0.79–0.88)	**2.07 × 10^−11^**	**2.20 × 10^−2^**	4.91	79.60	2.70 × 10^−2^
rs10811662	A	Total	METS-IR	0.74 (0.66–0.82)	8.32 × 10^−8^	0.75 (0.70–0.81)	**9.81 × 10^−15^**	0.84 (0.80–0.89)	**2.08 × 10^−10^**	**7.79 × 10^−3^**	4.59	78.20	3.20 × 10^−2^
rs10811662	A	Male	METS-VF	0.73 (0.60–0.91)	4.27 × 10^−3^	0.69 (0.62–0.78)	1.78 × 10^−10^	0.86 (0.80–0.92)	1.51 × 10^−5^	**7.48 × 10^−3^**	1.80	44.50	1.79 × 10^−1^
rs10811662	A	Male	METS-IR	0.71 (0.60–0.85)	1.89 × 10^−4^	0.74 (0.66–0.83)	1.11 × 10^−7^	0.85 (0.79–0.91)	1.02 × 10^−5^	**2.72 × 10^−2^**	3.09	67.60	7.90 × 10^−2^
rs10811662	A	Female	METS-VF	0.73 (0.65–0.82)	2.66 × 10^−7^	0.83 (0.76–0.90)	2.11 × 10^−5^	0.81 (0.75–0.88)	2.99 × 10^−7^	2.82 × 10^−1^	1.98	49.40	1.60 × 10^−1^
rs10811662	A	Female	METS-IR	0.75 (0.65–0.86)	5.83 × 10^−5^	0.76 (0.69–0.84)	**3.05 × 10^−8^**	0.84 (0.78–0.91)	4.89 × 10^−6^	9.57 × 10^−2^	2.10	52.30	1.48 × 10^−1^
rs183572120 (CPEB3)	A	Total	METS-VF	1.84 (1.49–2.26)	**9.33 × 10^−9^**	1.33 (1.14–1.56)	3.30 × 10^−4^	1.28 (1.12–1.46)	2.00 × 10^−4^	**1.43 × 10^−2^**	8.37	88.10	4.00 × 10^−3^
rs183572120	A	Total	METS-IR	1.70 (1.36–2.13)	3.91 × 10^−6^	1.45 (1.24–1.71)	5.79 × 10^−6^	1.25 (1.10–1.42)	7.75 × 10^−4^	**1.88 × 10^−2^**	5.58	82.10	1.80 × 10^−2^
rs183572120	A	Male	METS-VF	1.87 (1.23–2.84)	3.19 × 10^−3^	1.63 (1.27–2.08)	1.08 × 10^−4^	1.29 (1.08–1.53)	4.03 × 10^−3^	**4.79 × 10^−2^**	2.64	62.10	1.04 × 10^−1^
rs183572120	A	Male	METS-IR	1.87 (1.30–2.71)	8.04 × 10^−4^	1.53 (1.19–1.97)	8.75 × 10^−4^	1.27 (1.06–1.52)	8.35 × 10^−3^	**4.65 × 10^−2^**	3.42	70.70	6.50 × 10^−2^
rs183572120	A	Female	METS-VF	1.82 (1.44–2.32)	8.78 × 10^−7^	1.17 (0.95–1.44)	1.33 × 10^−1^	1.27 (1.04–1.55)	1.69 × 10^−2^	5.71 × 10^−2^	5.14	80.50	2.30 × 10^−2^
rs183572120	A	Female	METS-IR	1.61 (1.21–2.15)	1.08 × 10^−3^	1.39 (1.13–1.72)	2.11 × 10^−3^	1.22 (1.02–1.47)	3.13 × 10^−2^	1.29 × 10^−1^	2.56	61.00	1.10 × 10^−1^
rs191684511 (HHEX)	G	Total	METS-VF	2.21 (1.78–2.75)	**9.10 × 10^−13^**	1.41 (1.19–1.67)	8.68 × 10^−5^	1.44 (1.25–1.66)	4.81 × 10^−7^	**1.03 × 10^−2^**	10.53	90.50	1.00 × 10^−3^
rs191684511	G	Total	METS-IR	2.00 (1.57–2.54)	**1.70 × 10^−8^**	1.65 (1.39–1.96)	**9.24 × 10^−9^**	1.36 (1.18–1.56)	2.00 × 10^−5^	**7.88 × 10^−3^**	7.45	86.60	6.00 × 10^−3^
rs191684511	G	Male	METS-VF	1.95 (1.24–3.09)	4.14 × 10^−3^	1.65 (1.26–2.17)	2.91 × 10^−4^	1.44 (1.19–1.74)	1.42 × 10^−4^	1.86 × 10^−1^	1.47	31.90	2.26 × 10^−1^
rs191684511	G	Male	METS-IR	1.89 (1.25–2.87)	2.74 × 10^−3^	1.81 (1.39–2.35)	9.54 × 10^−6^	1.35 (1.11–1.64)	3.07 × 10^−3^	6.09 × 10^−2^	2.07	51.70	1.50 × 10^−1^
rs191684511	G	Female	METS-VF	2.29 (1.78–2.93)	**6.38 × 10^−11^**	1.27 (1.02–1.58)	3.30 × 10^−2^	1.46 (1.18–1.82)	6.17 × 10^−4^	**2.25 × 10^−2^**	7.07	85.80	8.00 × 10^−3^
rs191684511	G	Female	METS-IR	2.03 (1.51–2.72)	2.41 × 10^−6^	1.54 (1.23–1.93)	1.67 × 10^−4^	1.39 (1.14–1.69)	1.28 × 10^−3^	5.41 × 10^−2^	4.46	77.60	3.50 × 10^−2^
rs747219957 (CNNM2)	-	Total	METS-VF	0.82 (0.77–0.87)	**9.85 × 10^−10^**	0.95 (0.88–1.03)	1.93 × 10^−1^	0.90 (0.86–0.94)	7.60 × 10^−7^	7.46 × 10^−1^	0.00	0.00	9.48 × 10^−1^
rs747219957	-	Total	METS-IR	0.89 (0.84–0.94)	1.31 × 10^−4^	0.94 (0.86–1.02)	1.40 × 10^−1^	0.90 (0.86–0.94)	2.61 × 10^−6^	2.02 × 10^−1^	2.88	65.30	9.00 × 10^−2^
rs747219957	-	Male	METS-VF	0.85 (0.73–0.98)	2.83 × 10^−2^	1.04 (0.92–1.18)	5.39 × 10^−1^	0.90 (0.85–0.95)	2.21 × 10^−4^	1.67 × 10^−1^	5.77	82.70	1.60 × 10^−2^
rs747219957	-	Male	METS-IR	0.93 (0.83–1.04)	2.22 × 10^−1^	1.02 (0.90–1.15)	7.92 × 10^−1^	0.91 (0.85–0.96)	2.03 × 10^−3^	9.13 × 10^−1^	0.06	0.00	8.15 × 10^−1^
rs747219957	-	Female	METS-VF	0.81 (0.76–0.87)	**1.08 × 10^−8^**	0.89 (0.81–0.99)	3.17 × 10^−2^	0.90 (0.84–0.96)	8.34 × 10^−4^	3.56 × 10^−1^	1.59	36.90	2.08 × 10^−1^
rs747219957	-	Female	METS-IR	0.88 (0.82–0.94)	2.89 × 10^−4^	0.88 (0.79–0.99)	2.80 × 10^−2^	0.89 (0.84–0.95)	2.58 × 10^−4^	8.70 × 10^−2^	5.59	82.10	1.80 × 10^−2^
rs163184 (KCNQ1)	G	Total	METS-VF	1.36 (1.23–1.51)	**2.99 × 10^−9^**	1.22 (1.14–1.30)	**1.95 × 10^−8^**	1.18 (1.12–1.25)	**4.33 × 10^−10^**	**4.50 × 10^−2^**	5.70	82.50	1.70 × 10^−2^
rs163184	G	Total	METS-IR	1.19 (1.06–1.32)	1.94 × 10^−3^	1.24 (1.16–1.34)	**1.48 × 10^−9^**	1.21 (1.15–1.27)	**1.89 × 10^−12^**	9.04 × 10^−1^	0.09	0.00	7.64 × 10^−1^
rs163184	G	Male	METS-VF	1.41 (1.15–1.74)	1.09 × 10^−3^	1.27 (1.14–1.42)	1.81 × 10^−5^	1.20 (1.12–1.29)	2.53 × 10^−7^	1.64 × 10^−1^	2.05	51.20	1.52 × 10^−1^
rs163184	G	Male	METS-IR	1.24 (1.05–1.48)	1.31 × 10^−2^	1.23 (1.10–1.37)	1.76 × 10^−4^	1.24 (1.15–1.34)	**1.04 × 10^−8^**	8.86 × 10^−1^	0.00	0.00	1.00 × 10^0^
rs163184	G	Female	METS-VF	1.34 (1.20–1.51)	7.45 × 10^−7^	1.18 (1.08–1.29)	1.79 × 10^−4^	1.16 (1.07–1.26)	2.44 × 10^−4^	7.27 × 10^−2^	3.92	74.50	4.80 × 10^−2^
rs163184	G	Female	METS-IR	1.16 (1.01–1.33)	3.81 × 10^−2^	1.25 (1.14–1.37)	3.06 × 10^−6^	1.18 (1.09–1.27)	1.56 × 10^−5^	9.28 × 10^−1^	0.05	0.00	8.31 × 10^−1^
rs671 (ALDH2)	A	Total	METS-VF	0.90 (0.78–1.04)	1.49 × 10^−1^	0.84 (0.76–0.92)	3.86 × 10^−4^	0.75 (0.69–0.82)	**8.34 × 10^−12^**	**3.69 × 10^−4^**	4.35	77.00	3.70 × 10^−2^
rs671	A	Total	METS-IR	0.83 (0.71–0.97)	2.27 × 10^−2^	0.88 (0.79–0.97)	1.23 × 10^−2^	0.74 (0.69–0.80)	**1.86 × 10^−13^**	**1.64 × 10^−3^**	1.68	40.30	1.95 × 10^−1^
rs671	A	Male	METS-VF	0.60 (0.43–0.83)	2.06 × 10^−3^	0.65 (0.55–0.77)	9.22 × 10^−7^	0.68 (0.61–0.76)	**3.41 × 10^−11^**	6.67 × 10^−1^	0.51	0.00	4.77 × 10^−1^
rs671	A	Male	METS-IR	0.61 (0.46–0.81)	4.70 × 10^−4^	0.69 (0.59–0.82)	2.97 × 10^−5^	0.65 (0.57–0.73)	**9.50 × 10^−13^**	2.80 × 10^−1^	0.13	0.00	7.24 × 10^−1^
rs671	A	Female	METS-VF	1.02 (0.86–1.20)	8.41 × 10^−1^	0.97 (0.86–1.10)	6.37 × 10^−1^	0.87 (0.77–0.97)	1.55 × 10^−2^	**4.89 × 10^−2^**	2.51	60.20	1.13 × 10^−1^
rs671	A	Female	METS-IR	1.00 (0.83–1.21)	9.83 × 10^−1^	1.03 (0.90–1.17)	6.56 × 10^−1^	0.86 (0.77–0.95)	4.32 × 10^−3^	**3.42 × 10^−2^**	2.01	50.20	1.56 × 10^−1^
rs11658063 (HNF1B)	C	Total	METS-VF	1.25 (1.13–1.39)	3.61 × 10^−5^	1.08 (1.00–1.16)	4.62 × 10^−2^	1.20 (1.13–1.27)	**5.07 × 10^−10^**	5.94 × 10^−1^	0.53	0.00	4.66 × 10^−1^
rs11658063	C	Total	METS-IR	1.23 (1.10–1.38)	2.95 × 10^−4^	1.20 (1.11–1.29)	2.10 × 10^−6^	1.14 (1.07–1.20)	1.16 × 10^−5^	2.10 × 10^−1^	1.65	39.20	2.00 × 10^−1^
rs11658063	C	Male	METS-VF	1.15 (0.92–1.44)	2.34 × 10^−1^	1.04 (0.93–1.17)	4.65 × 10^−1^	1.19 (1.10–1.28)	6.33 × 10^−6^	1.53 × 10^−1^	0.09	0.00	7.67 × 10^−1^
rs11658063	C	Male	METS-IR	1.19 (0.99–1.44)	6.08 × 10^−2^	1.15 (1.02–1.29)	2.17 × 10^−2^	1.14 (1.05–1.24)	1.09 × 10^−3^	8.22 × 10^−1^	0.19	0.00	6.64 × 10^−1^
rs11658063	C	Female	METS-VF	1.28 (1.13–1.45)	6.58 × 10^−5^	1.10 (1.00–1.21)	5.45 × 10^−2^	1.20 (1.10–1.31)	2.71 × 10^−5^	7.77 × 10^−1^	0.67	0.00	4.14 × 10^−1^
rs11658063	C	Female	METS-IR	1.25 (1.08–1.45)	2.07 × 10^−3^	1.24 (1.12–1.37)	1.94 × 10^−5^	1.12 (1.03–1.22)	4.99 × 10^−3^	1.33 × 10^−1^	1.73	42.20	1.88 × 10^−1^
rs12625671 (HNF4A)	C	Total	METS-VF	1.09 (0.98–1.20)	1.06 × 10^−1^	1.08 (1.01–1.16)	2.09 × 10^−2^	1.16 (1.10–1.23)	**1.61 × 10^−8^**	9.39 × 10^−2^	1.31	23.50	2.53 × 10^−1^
rs12625671	C	Total	METS-IR	1.09 (0.98–1.22)	9.40 × 10^−2^	1.15 (1.07–1.23)	1.05 × 10^−4^	1.12 (1.06–1.18)	2.46 × 10^−5^	8.37 × 10^−1^	0.12	0.00	7.30 × 10^−1^
rs12625671	C	Male	METS-VF	0.83 (0.68–1.03)	8.56 × 10^−2^	1.05 (0.94–1.17)	3.91 × 10^−1^	1.20 (1.11–1.28)	4.78 × 10^−7^	**4.28 × 10^−4^**	10.39	90.40	1.00 × 10^−3^
rs12625671	C	Male	METS-IR	0.98 (0.83–1.17)	8.38 × 10^−1^	1.11 (1.00–1.23)	6.03 × 10^−2^	1.16 (1.08–1.25)	8.22 × 10^−5^	8.06 × 10^−2^	3.03	66.90	8.20 × 10^−2^
rs12625671	C	Female	METS-VF	1.19 (1.05–1.33)	4.40 × 10^−3^	1.10 (1.01–1.20)	2.78 × 10^−2^	1.12 (1.03–1.21)	4.93 × 10^−3^	6.10 × 10^−1^	0.59	0.00	4.41 × 10^−1^
rs12625671	C	Female	METS-IR	1.16 (1.02–1.33)	2.87 × 10^−2^	1.18 (1.08–1.30)	4.50 × 10^−4^	1.08 (1.00–1.16)	4.39 × 10^−2^	2.09 × 10^−1^	0.93	0.00	3.35 × 10^−1^

Note: OR, odds ratio; CI, confidence interval; Q, Cochran’s Q statistic; I^2^, heterogeneity statistic. Interaction *p*-values represent tests for differences in genetic associations across metabolic tertiles. Heterogeneity was assessed using Cochran’s Q test and the I^2^ statistic. The number of T2DM cases and controls included in each GWAS stratum, including sex-stratified analyses, are provided in Supplementary Table S1. Genomic coordinates are based on the GRCh37/hg19 reference genome.

## Data Availability

The datasets analyzed in the current study are available from the Korean Genome and Epidemiology Study (KoGES) repository upon reasonable request and approval from the Korea Disease Control and Prevention Agency (KDCA). Restrictions apply to the availability of these data, which were used under license for the current study.

## Supplementary Materials

The following supporting information can be downloaded at: https://www.mdpi.com/article/10.3390/ijms27219844/s1.
