# Supplementary material for: Genetic Associations with Type 2 Diabetes Differ According to Metabolic Contexts Defined by Insulin Resistance and Visceral Adiposity
Source: Int J Mol Sci. 2026 Nov 3;27(21):9844. doi: 10.3390/ijms27219844 (PMC13763703; doi:10.3390/ijms27219844)
Supplement: Supplementary file 1 [file ijms-27-09844-s001.zip › ijms-4612639-supplementary.pdf]

Supplementary Table S1. Numbers of T2DM cases and controls across METS-IR and METS-VF tertiles, stratified by sex.

(A) METS-IR stratification: T1, T2, and T3 tertiles, including the total population, men, and women.

METS-IR Tertile Group	Stratified Group	Total	w Type2 Diabetes	w/o Type2 Diabetes
T1	Total (n, %)	13583	764 (5.62)	12,819 (94.38)
	Male (n, %)	3068	312 (10.17)	2756 (89.83)
	Female (n, %)	10515	452 (4.30)	10,063 (95.70)
T2	Total (n, %)	13043	1929 (14.79)	11114 (85.21)
	Male (n, %)	4851	857 (17.67)	3994 (82.33)
	Female (n, %)	8192	1072	7120
T3	Total (n, %)	14344	4351 (30.33)	9993 (69.67)
	Male (n, %)	7117	2254 (31.67)	4863 (68.33)
	Female (n, %)	7227	2097 (29.02)	5130 (70.98)

(B) METS-VF stratification: T1, T2, and T3 tertiles, including the total population, men, and women.

METS-VF Tertile Group	Stratified Group	Total	w Type2 Diabetes	w/o Type2 Diabetes
T1	Total (n, %)	13856	837 (6.04)	13,019 (93.96)
	Male (n, %)	2375	213 (8.97)	2162 (91.03)
	Female (n, %)	11481	624 (5.44)	10,857 (94.56)
T2	Total (n, %)	13313	2045 (15.36)	11268 (84.64)
	Male (n, %)	4896	820 (16.75)	4076 (83.25)
	Female (n, %)	8417	1225 (14.55)	7192 (85.45)
T3	Total (n, %)	13775	4155 (30.16)	9620 (69.84)

	Male (n, %)	7757	2386 (30.76)	5371 (69.24)
	Female (n, %)	6018	1769 (29.40)	4249 (70.60)

Supplementary Table S2. Genomic annotations and Minor allele frequencies of lead SNPs across populations.

Gene	SNP	Chr	Bp	Ref	Alt	Consequence	Minor Allele	Minor Allele Frequency			
								This Study	Other Ethnics		
									EAS	EUR	AMR
CDKAL1	rs9368219	6	20674691	C	T	Intronic	T	0.46	0.38	0.18	0.18
PAX4	rs2233580	7	127253550	C	T	Missense variant	T	0.07	0.10	0.00	0.00
DNAJC5B-TRIM55	rs1304925012	8	67026314	A	-	Intergenic	-	0.02	0.00	0.00	0.00
SLC30A8	rs3802177	8	118185025	G	A	3' prime UTR	A	0.41	0.46	0.28	0.27
CDKN2A/B	rs10811662	9	22134253	G	A	Intergenic	A	0.44	0.44	0.17	0.13
CPEB3	rs183572120	10	93907282	G	A	Intronic	A	0.04	0.01	0.00	0.00
HHEX	rs191684511	10	94452672	C	G	Intronic	G	0.03	0.00	0.00	0.00
CNNM2	rs747219957	10	104778443	insT	-	Intronic	-	0.25	0.29	0.09	0.20
KCNQ1	rs163184	11	2847069	T	G	Intronic	G	0.41	0.42	0.49	0.40
ALDH2	rs671	12	112241766	G	A	Missense variant	A	0.16	0.17	0.00	0.00
HNF1B	rs11658063	17	36103872	C	G	Intronic	C	0.30	0.23	0.39	0.29
HNF4A	rs12625671	20	42994812	T	C	Intronic	C	0.44	0.41	0.12	0.42

Supplementary Table S3. Coded allele frequencies of lead SNPs across METS-VF and METS-IR tertiles, stratified by sex.

SNP	Coded allele	METS-VF									METS-IR								
		Total			Male			Female			Total			Male			Female		
		T1	T2	T3	T1	T2	T3	T1	T2	T3	T1	T2	T3	T1	T2	T3	T1	T2	T3
rs9368219	T	0.47	0.47	0.46	0.48	0.48	0.46	0.47	0.47	0.47	0.47	0.47	0.47	0.47	0.48	0.46	0.47	0.47	0.48
rs2233580	T	0.07	0.07	0.08	0.08	0.07	0.08	0.07	0.07	0.07	0.07	0.07	0.07	0.08	0.08	0.08	0.07	0.07	0.07
rs1304925012	G	0.01	0.01	0.02	0.02	0.02	0.01	0.01	0.01	0.02	0.01	0.02	0.02	0.02	0.02	0.01	0.01	0.01	0.02
rs3802177	A	0.41	0.41	0.41	0.41	0.41	0.41	0.41	0.40	0.41	0.41	0.41	0.41	0.40	0.41	0.41	0.41	0.41	0.41
rs10811662	A	0.43	0.43	0.44	0.45	0.42	0.43	0.43	0.44	0.44	0.44	0.43	0.43	0.44	0.44	0.43	0.44	0.43	0.43
rs183572120	A	0.04	0.04	0.04	0.04	0.04	0.04	0.04	0.04	0.04	0.04	0.04	0.04	0.04	0.04	0.04	0.04	0.04	0.04
rs191684511	G	0.03	0.03	0.03	0.03	0.03	0.03	0.03	0.04	0.03	0.03	0.03	0.03	0.03	0.03	0.03	0.03	0.03	0.03
rs747219957	C	0.24	0.25	0.25	0.23	0.25	0.25	0.24	0.24	0.25	0.25	0.24	0.25	0.24	0.24	0.25	0.25	0.24	0.24
rs163184	G	0.42	0.42	0.41	0.42	0.43	0.41	0.42	0.42	0.41	0.41	0.42	0.42	0.42	0.42	0.42	0.41	0.42	0.42
rs671	A	0.16	0.16	0.15	0.18	0.16	0.15	0.16	0.16	0.15	0.16	0.16	0.15	0.16	0.16	0.15	0.16	0.15	0.15
rs11658063	C	0.30	0.30	0.30	0.29	0.30	0.29	0.31	0.30	0.30	0.30	0.30	0.30	0.29	0.30	0.30	0.30	0.30	0.30
rs12625671	C	0.45	0.45	0.44	0.45	0.45	0.45	0.45	0.45	0.44	0.45	0.44	0.45	0.45	0.44	0.45	0.45	0.45	0.45

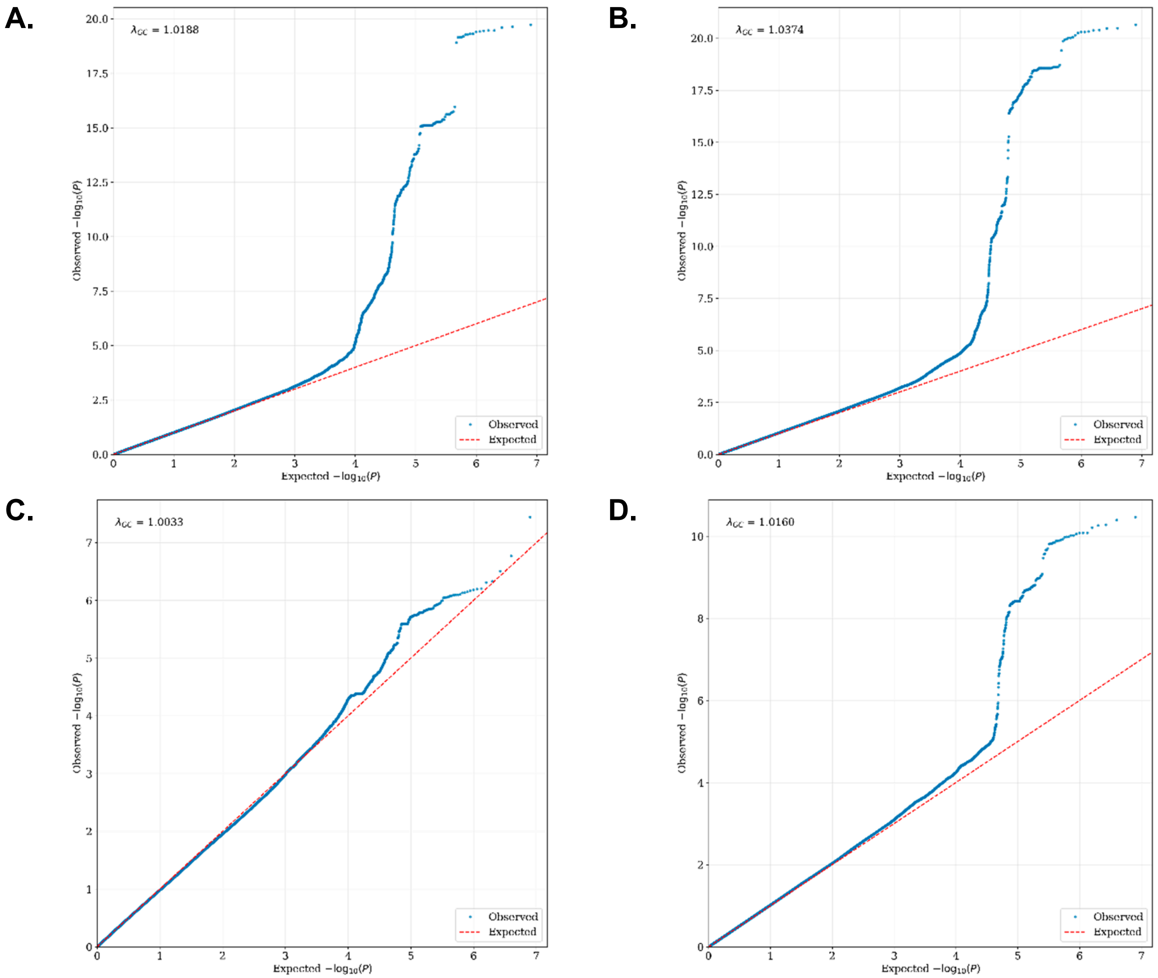
A.
λ_{GC} = 1.0188
Observed $-\log_{10}(P)$
Expected $-\log_{10}(P)$
Observed
Expected
B.
λ_{GC} = 1.0374
Observed $-\log_{10}(P)$
Expected $-\log_{10}(P)$
Observed
Expected
C.
λ_{GC} = 1.0033
Observed $-\log_{10}(P)$
Expected $-\log_{10}(P)$
Observed
Expected
D.
λ_{GC} = 1.0160
Observed $-\log_{10}(P)$
Expected $-\log_{10}(P)$
Observed
Expected

E

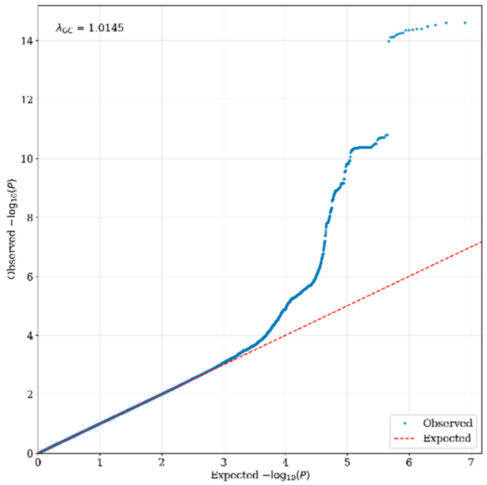

F

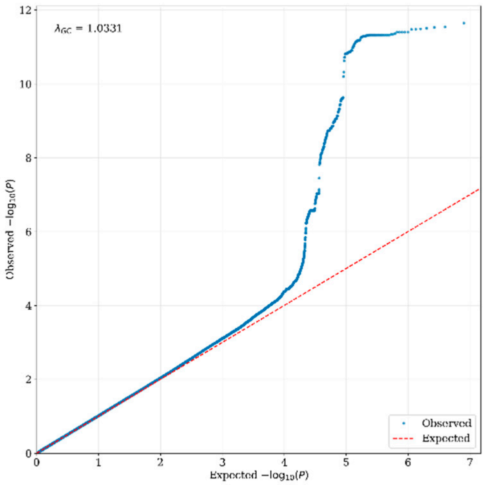

Supplementary Figure S1. Quantile–quantile plots of GWAS association P values for diabetes prevalence across METS-VF tertiles and sex strata. Panels show results for (A) T1 in the total population, (B) T3 in the total population, (C) T1 in males, (D) T3 in males, (E) T1 in females, and (F) T3 in females. Blue points represent observed $-\log_{10}(P)$ values, and red dashed lines indicate the expected distribution under the null hypothesis. The genomic inflation factor (λGC) is displayed in each panel.

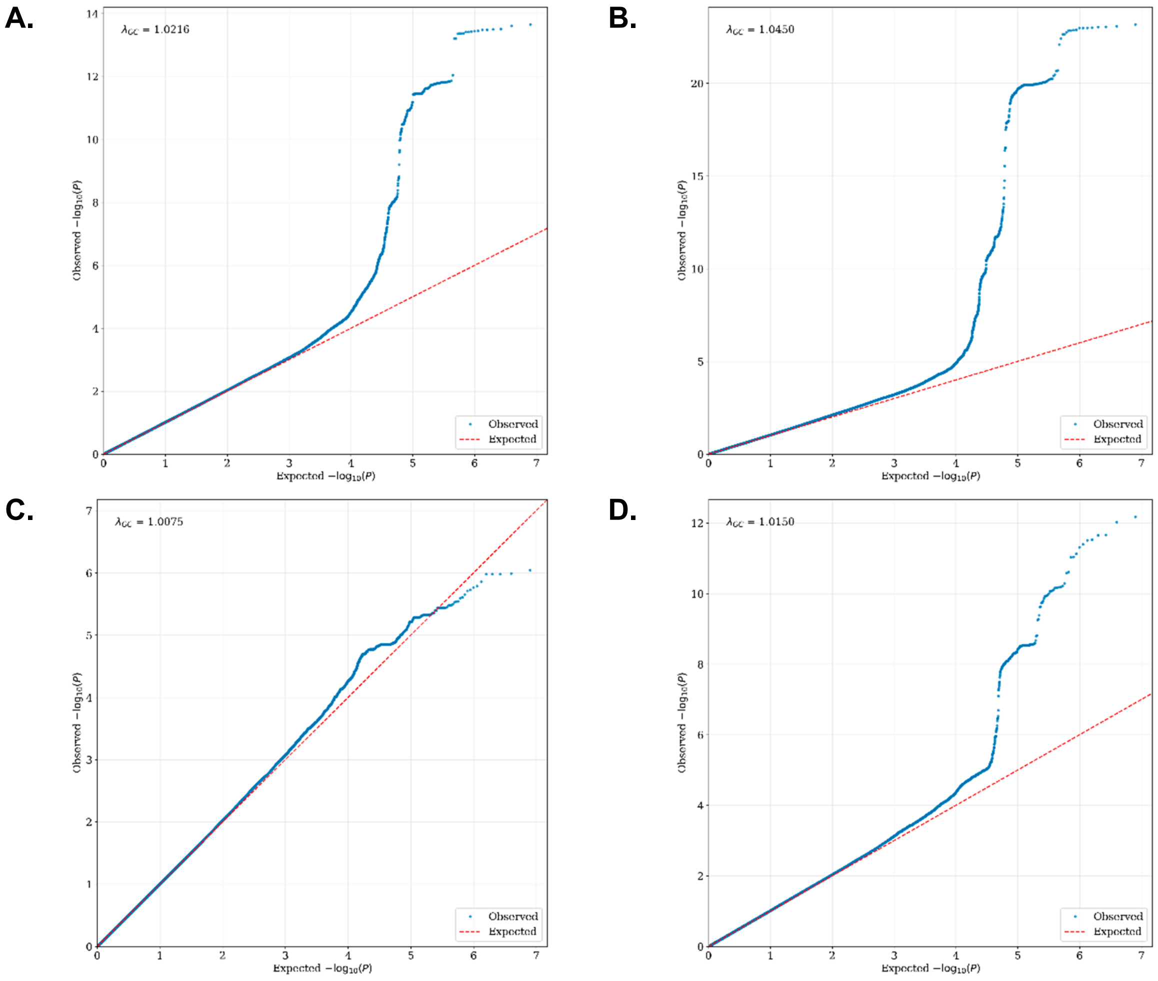
A.
λ_{GC} = 1.0216
B.
λ_{GC} = 1.0450
C.
λ_{GC} = 1.0075
D.
λ_{GC} = 1.0150
Observed
Expected
Observed $-\log_{10}(P)$
Expected $-\log_{10}(P)$

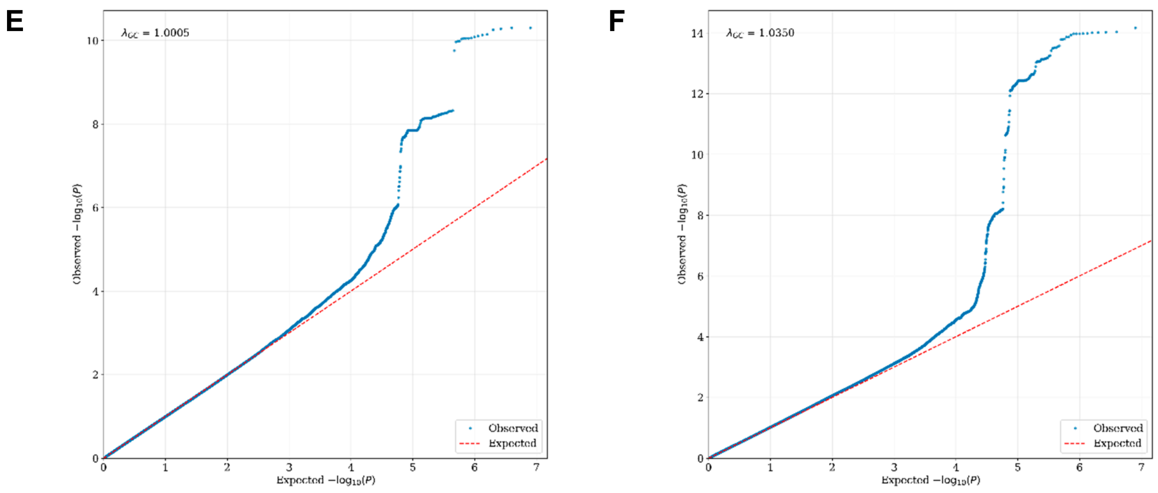

Supplementary Figure S2. Quantile–quantile plots of GWAS association P values for diabetes prevalence across METS-IR tertiles and sex strata. Panels show results for (A) T1 in the total population, (B) T3 in the total population, (C) T1 in males, (D) T3 in males, (E) T1 in females, and (F) T3 in females. Blue points represent observed $-\log_{10}$(P) values, and red dashed lines indicate the expected distribution under the null hypothesis. The genomic inflation factor (λGC) is displayed in each panel.

Supplementary Figure S3. Regional association plots of five selected type 2 diabetes-associated loci across METS-VF and METS-IR strata.

A. CDKAL1 (rs9368219)

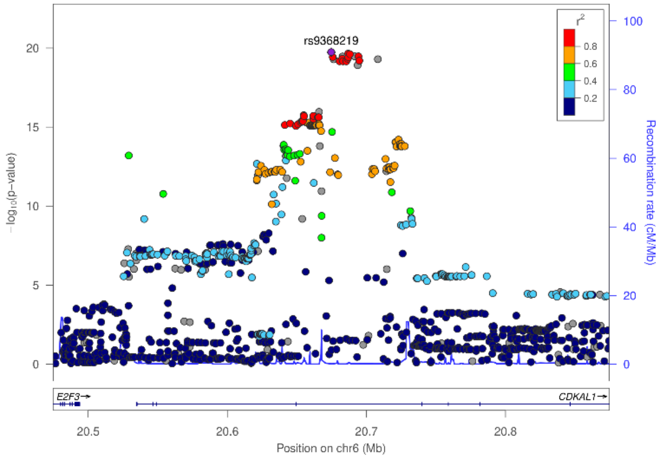

Diabetes.METS-VF(T1) in Total.rs9368219

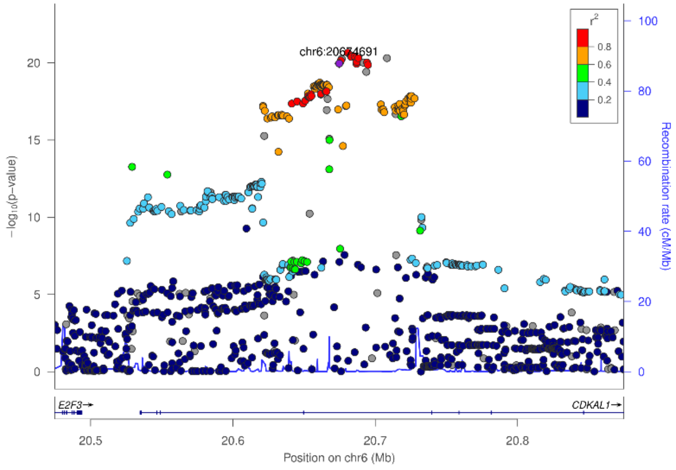

Diabetes.METS-VF(T3) in Total.rs9368219

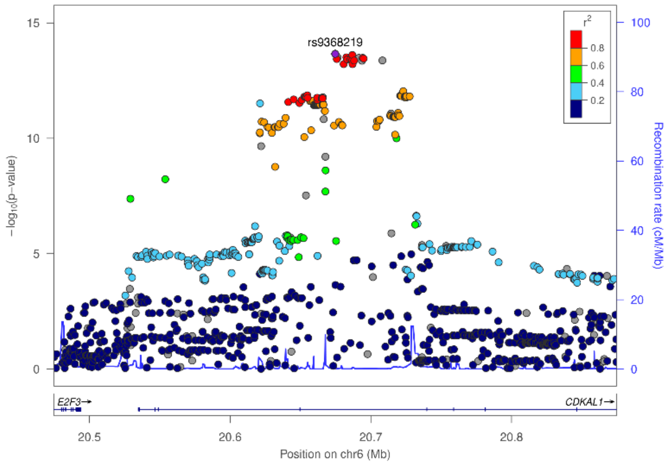

Diabetes.METS-IR(T1) in Total.rs9368219

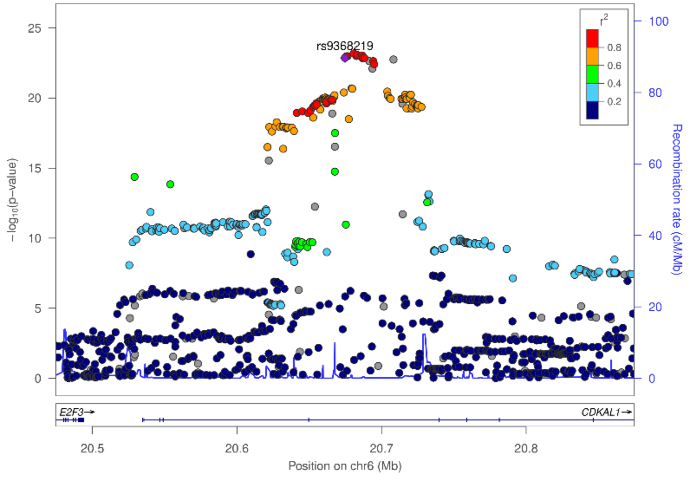

Diabetes.METS-IR(T3) in Total.rs9368219

B. PAX4 (rs2233580)

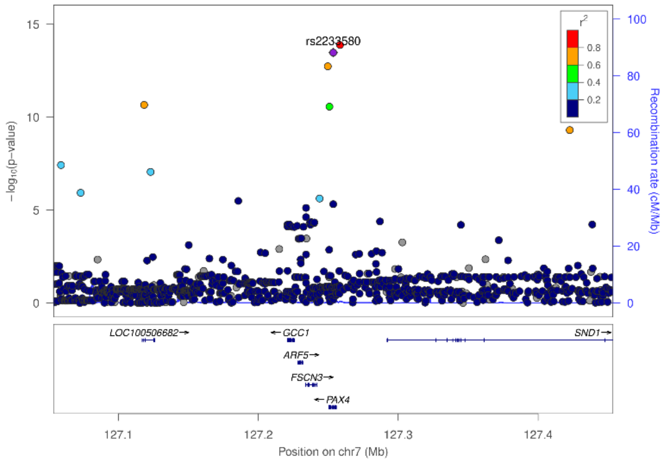

Diabetes.METS-VF(T1) in Total.rs2233580

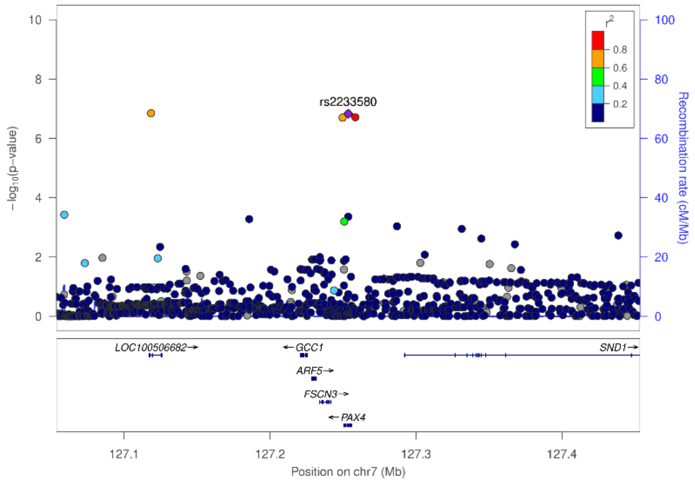

Diabetes.METS-VF(T3) in Total.rs2233580

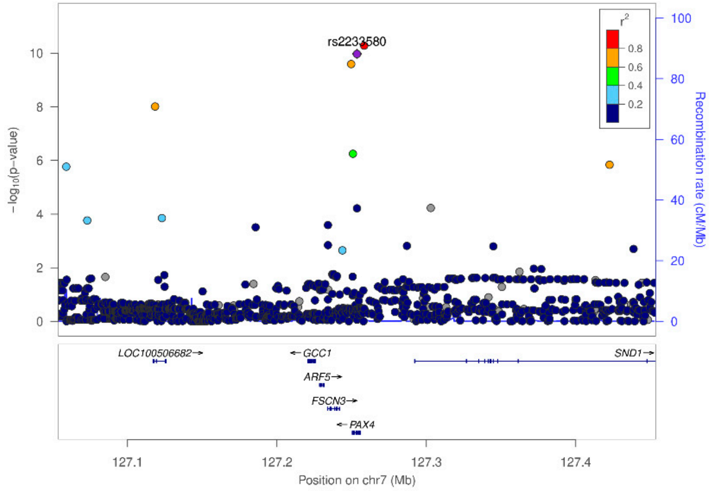

Diabetes.METS-IR(T1) in Total.rs2233580

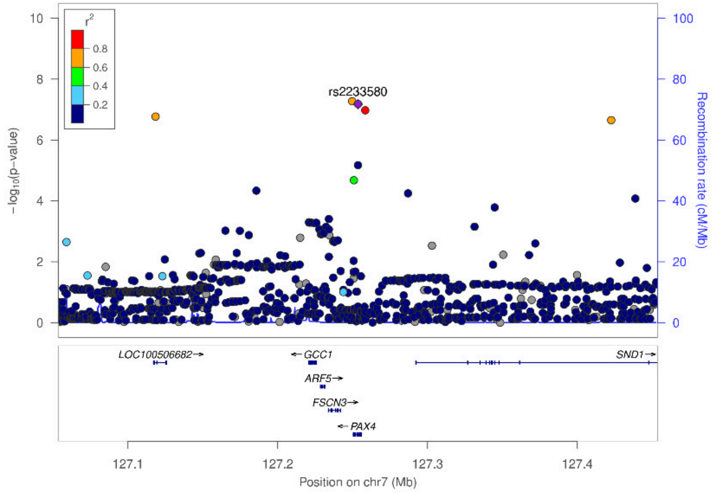

Diabetes.METS-IR(T3) in Total.rs2233580

C. CDKN2A/B (rs10811662)

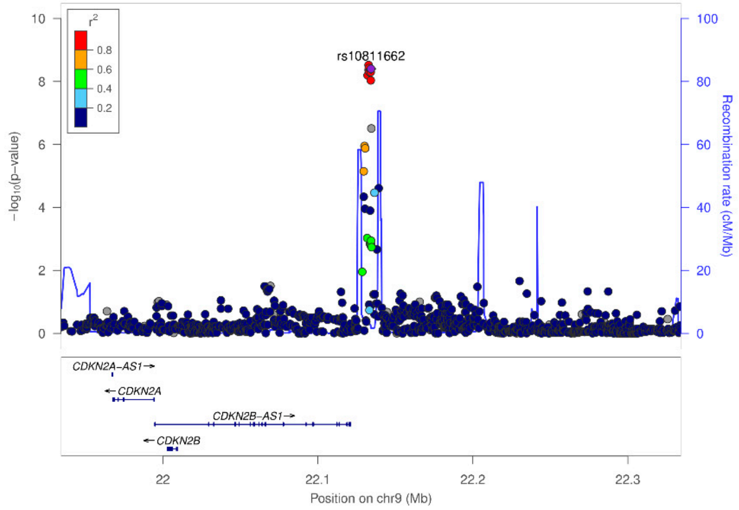

Diabetes.METS-VF(T1) in Total.rs10811662

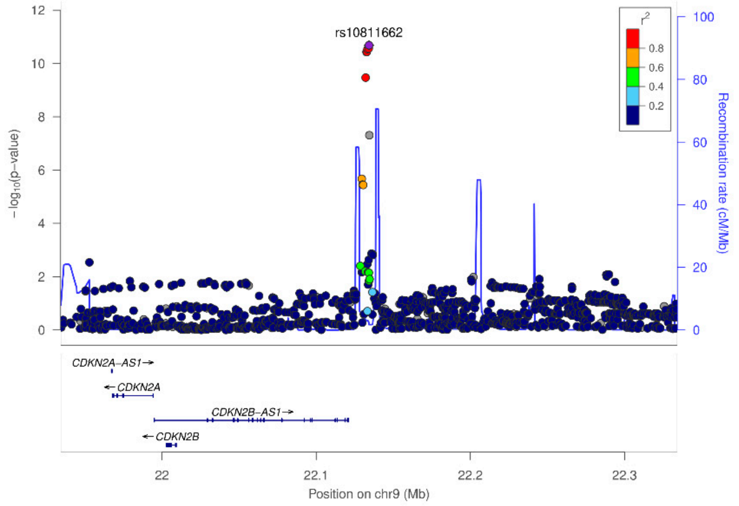

Diabetes.METS-VF(T3) in Total.rs10811662

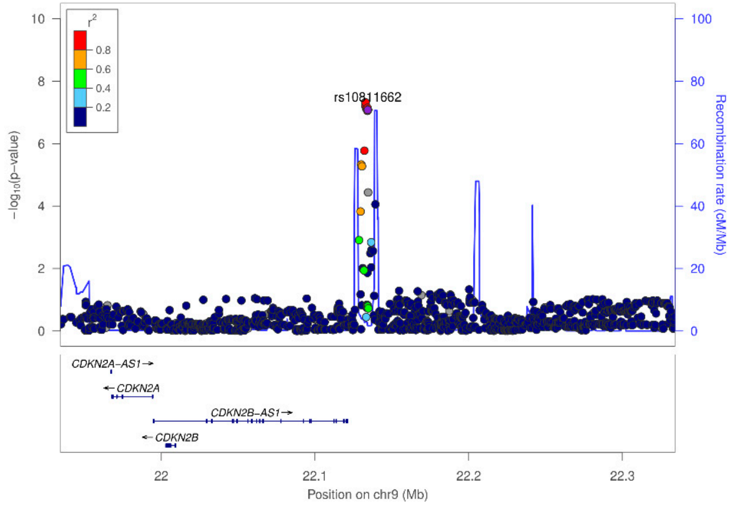

Diabetes.METS-IR(T1) in Total.rs10811662

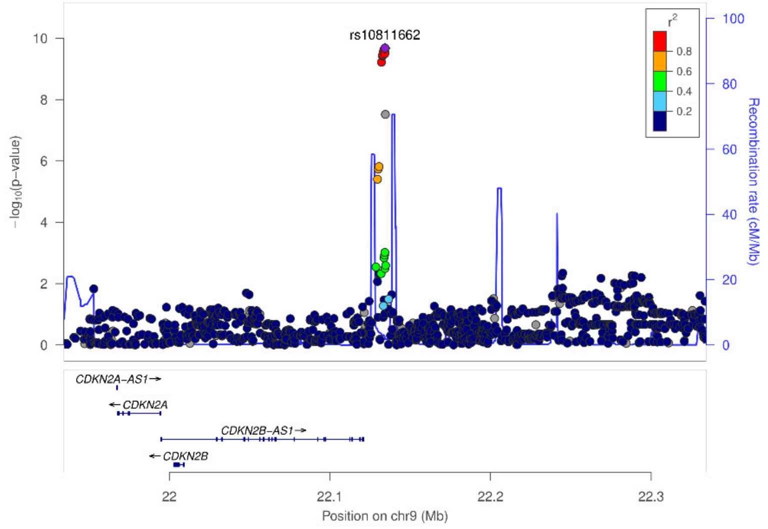

Diabetes.METS-IR(T3) in Total.rs10811662

D. HHEX (rs191684511)

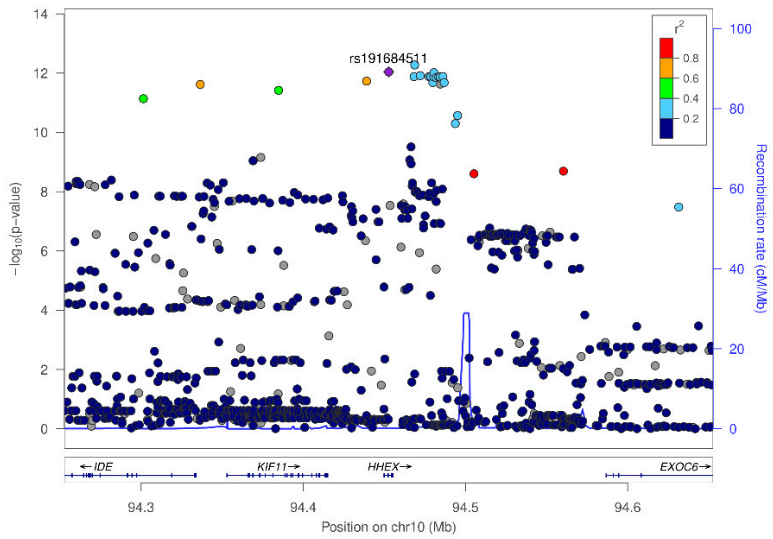

Diabetes.METS-VF(T1) in Total.rs191684511

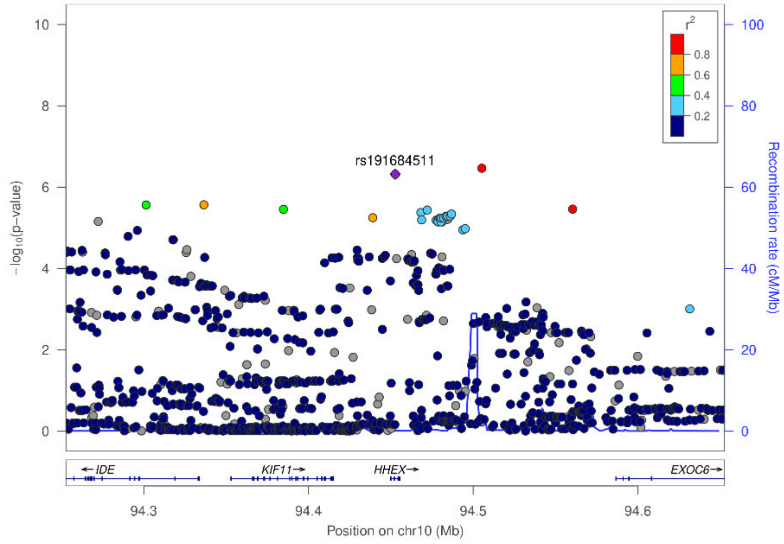

Diabetes.METS-VF(T3) in Total.rs191684511

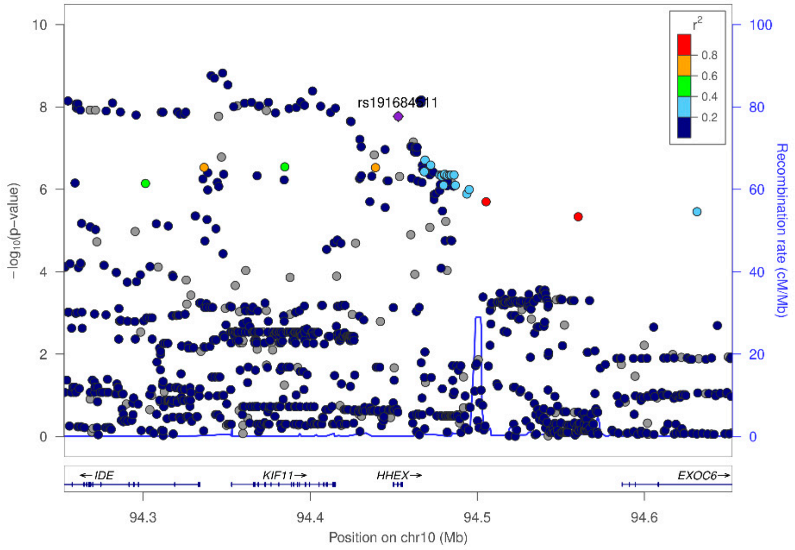

Diabetes.METS-IR(T1) in Total.rs191684511

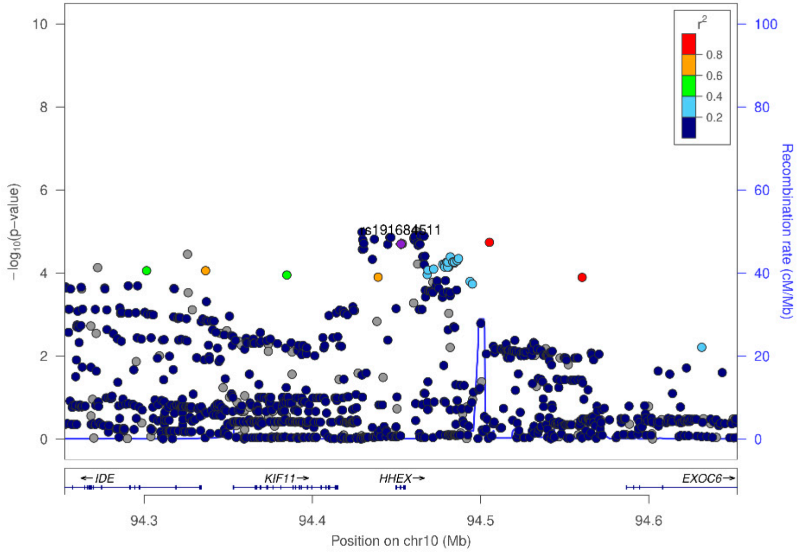

Diabetes.METS-IR(T3) in Total.rs191684511

E. CPEB3 (rs183572120)

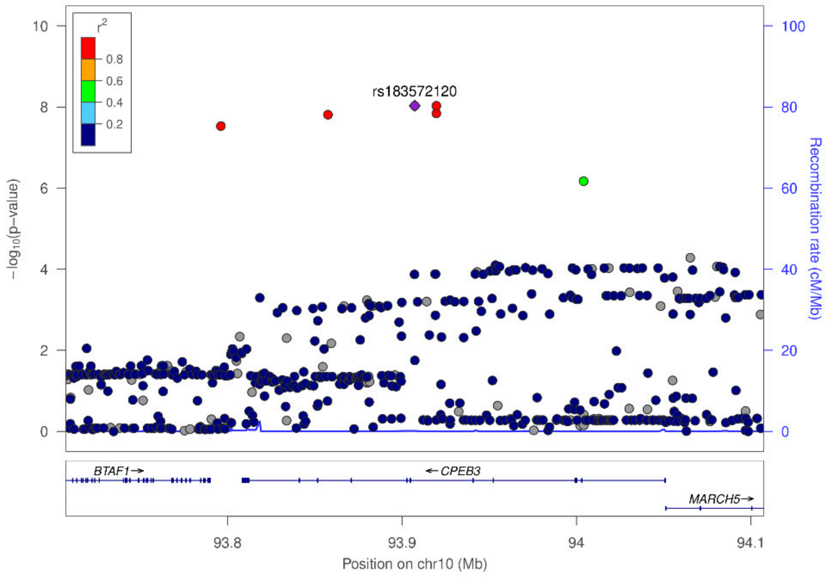

Diabetes.METS-VF(T1) in Total.rs183572120

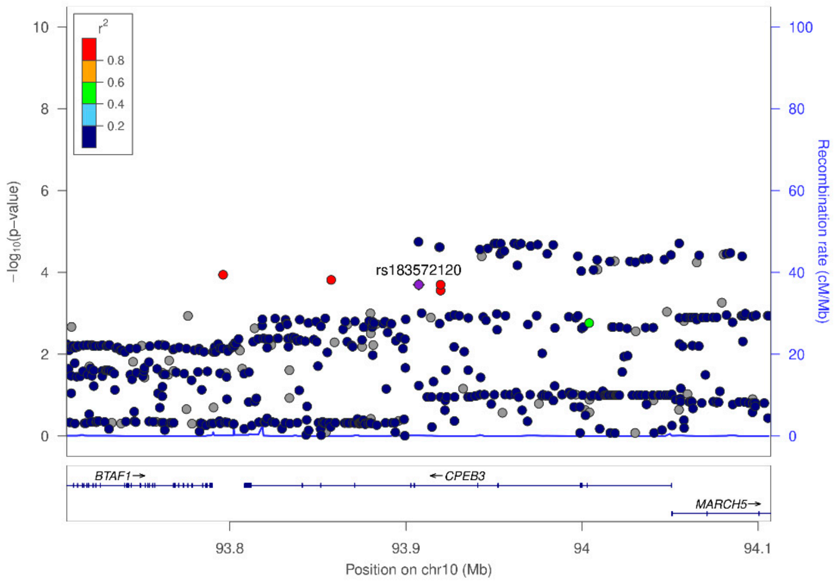

Diabetes.METS-VF(T3) in Total.rs183572120

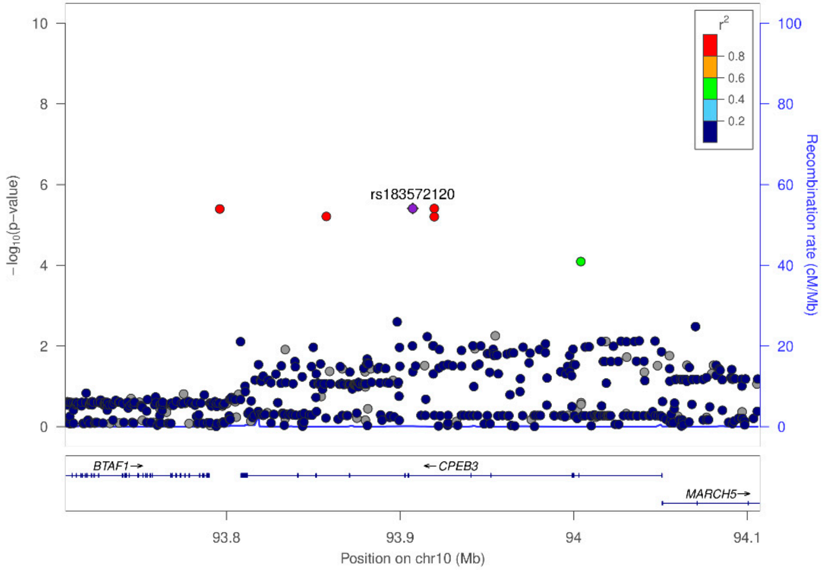

Diabetes.METS-IR(T1) in Total.rs183572120

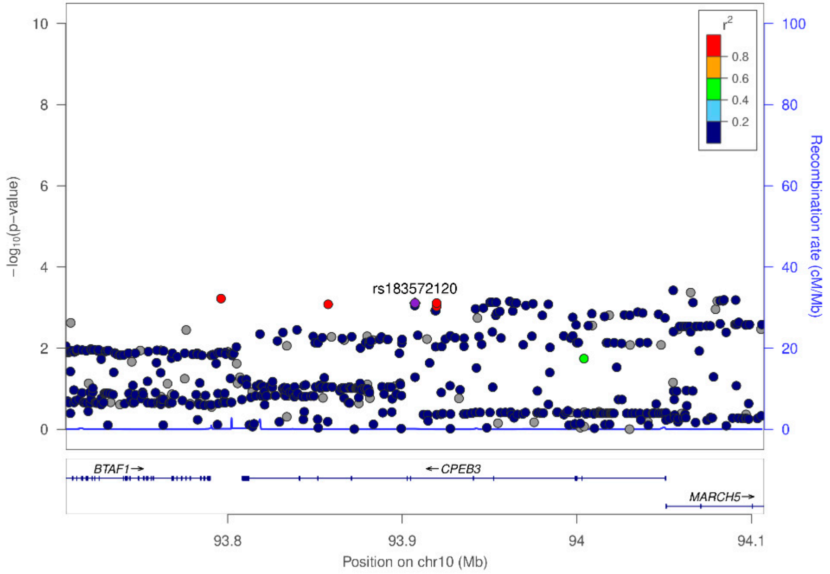

Diabetes.METS-IR(T3) in Total.rs183572120

Supplementary Figure S3. Regional association plots of five selected type 2 diabetes-associated loci across METS-VF and METS-IR strata.

Regional association plots are shown for (A) CDKAL1 (rs9368219), (B) PAX4 (rs2233580), (C) CDKN2A/B (rs10811662), (D) HHEX (rs191684511), and (E) CPEB3 (rs183572120). For each locus, four plots are presented in the following order: METS-VF T1, METS-VF T3, METS-IR T1, and METS-IR T3. All analyses were performed in the total population. T1 and T3 represent the lowest and highest tertiles of the corresponding metabolic index, respectively. Regional association plots illustrate the distribution of genetic association signals surrounding the selected loci.
